# Endoplasmic reticulum stress: molecular mechanism and therapeutic targets

**DOI:** 10.1038/s41392-023-01570-w

**Published:** 2023-09-15

**Authors:** Xingyi Chen, Chaoran Shi, Meihui He, Siqi Xiong, Xiaobo Xia

**Affiliations:** 1 Eye Center of Xiangya Hospital, Central South University, 410008 Changsha, Hunan China; 2 Hunan Key Laboratory of Ophthalmology, Central South University, 410008 Changsha, China; 3 National Clinical Research Center for Geriatric Disorders, Xiangya Hospital, Central South University, Changsha, China

**Keywords:** Molecular neuroscience, Cell death in the nervous system, Neurological disorders

## Abstract

The endoplasmic reticulum (ER) functions as a quality-control organelle for protein homeostasis, or “proteostasis”. The protein quality control systems involve ER-associated degradation, protein chaperons, and autophagy. ER stress is activated when proteostasis is broken with an accumulation of misfolded and unfolded proteins in the ER. ER stress activates an adaptive unfolded protein response to restore proteostasis by initiating protein kinase R-like ER kinase, activating transcription factor 6, and inositol requiring enzyme 1. ER stress is multifaceted, and acts on aspects at the epigenetic level, including transcription and protein processing. Accumulated data indicates its key role in protein homeostasis and other diverse functions involved in various ocular diseases, such as glaucoma, diabetic retinopathy, age-related macular degeneration, retinitis pigmentosa, achromatopsia, cataracts, ocular tumors, ocular surface diseases, and myopia. This review summarizes the molecular mechanisms underlying the aforementioned ocular diseases from an ER stress perspective. Drugs (chemicals, neurotrophic factors, and nanoparticles), gene therapy, and stem cell therapy are used to treat ocular diseases by alleviating ER stress. We delineate the advancement of therapy targeting ER stress to provide new treatment strategies for ocular diseases.

## Introduction

Proteostasis is fundamental to cell survival, and an imbalance will result in diseases including metabolic, neurodegenerative, oncological, and cardiovascular disorders.^1^ The endoplasmic reticulum (ER) functions as a quality-control organelle for the proteins it produces, allowing only normal proteins to exit its vesicles.^2^ Protein quality control systems are responsible for maintaining proteostasis, including chaperones, ATPases, glucose-regulated protein 94 (Grp94), BiP (an Hsp70 family member), and two proteolytic systems: the ubiquitin–proteasome and the lysosome–autophagy systems.^3^ Misfolded proteins produced under stress conditions are then removed from the folding machinery, relocated from the ER into the cytosol, and degraded in the ubiquitin-proteasome via a series of pathways collectively referred to as ER-associated degradation (ERAD).^4,5^ However, persistent misfolded proteins will accumulate and lead to ER stress, which can elicit an adaptive response called the unfolded protein response (UPR). The consequences of UPR include protein synthesis inhibition, regulation of gene expression,^6,7^ and cell fate decisions like apoptosis^8^ to meet the cell’s demands.

**Fig. 1:**
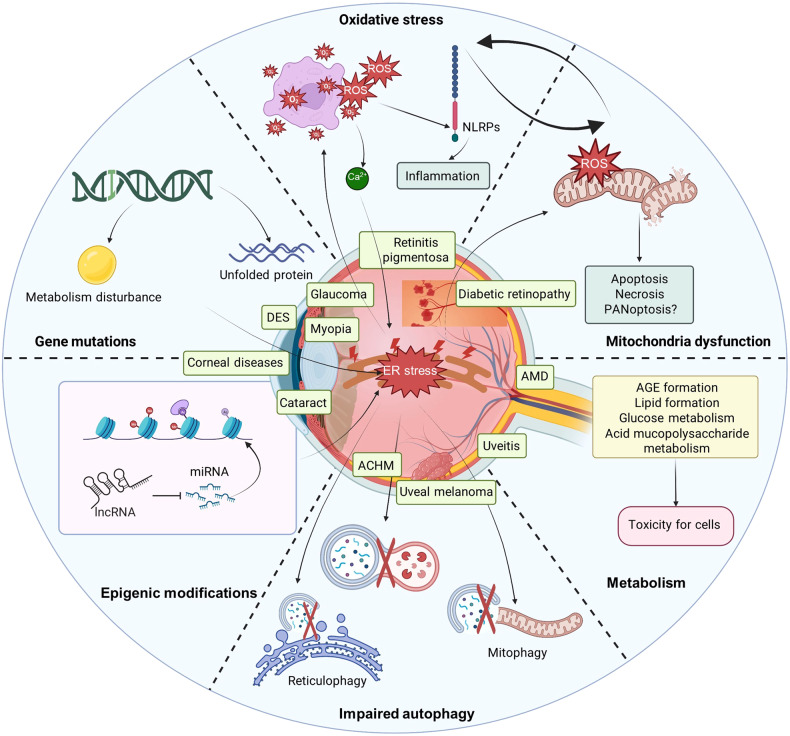
Overview of the regulatory mechanisms of ER stress in ocular diseases. ER stress plays an important role in several ocular diseases, including glaucoma, diabetic retinopathy, age-related macular dystrophy (AMD), retinitis pigmentosa (RP), achromatopsia (ACHM), cataract, corneal diseases, DES, myopia, uveitis, and uveal melanoma. The concrete mechanisms of ER stress in ocular diseases include regulation of gene mutations, epigenic modifications, impaired autophagy, oxidative stress, mitochondria dysfunction, and metabolism. The figure was created with BioRender.com (https://www.biorender.com/). AMD age-related macular dystrophy, RP retinitis pigmentosa, ACHM achromatopsia, DES dry eye syndrome

Increasing evidence indicates that ER stress is crucial in the pathology of ocular diseases (Fig. 1). Proteostasis imbalance-induced ER stress and its related mutations have been broadly reported in the field of ophthalmology, making them a promising treatment target in ocular diseases. In this review, we will discuss the latest advances, focusing on the molecular mechanisms and therapeutic targets between ER stress and ocular diseases.

## Unfolded protein response

**Fig. 2:**
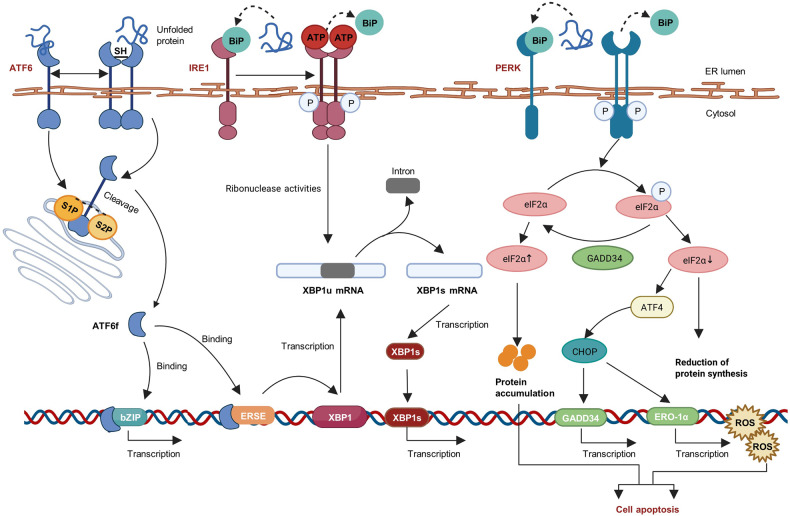
Unfolded protein response signaling pathways. Accumulation of misfolded and unfolded protein in endoplasmic reticulum (ER) will replace the BiP binding on PERK, ATF6 and IRE1, and activate them. PERK causes the phosphorylation of eIF2α, which leads to a reduction of ER protein accumulation and translation of the ATF4 mRNA. ATF4 then interacts with CHOP, which controls the expression of the target genes, such as GADD34 and ERO-1α. GADD34 encodes a regulatory subunit of an eIF2α-directed phosphatase complex, which in turn dephosphorylates eIF2α and recovers protein synthesis. The consequence of PERK pathway can be cell apoptosis. Under ER stress, IRE1 becomes dimerized and activated. The activated IRE1 excises an intron from XBP1 and transforms it into spliced XBP1 (XBP1s). XBP1s is transported to the nucleus, where it facilitates gene translation. Facing ER stress, ATF6 is transported to the Golgi apparatus and cleaved by Site-1 (S1P) and Site-2 (S2P) proteases. After the cleavage, ATF6 releases a cytosolic fragment (ATF6f), which directly controls the genes encoding ERAD components such as the basic transcription of leucine zipper (bZip) family and XBP1. The figure was created with BioRender.com (https://www.biorender.com/). ATF4 activating transcription factor 4, eukaryotic translation initiation factor 2α (eIF2α), C/EBP-homologous protein (CHOP), PERK PKR-like ER kinase, ATF6 activating transcription factor 6, IRE1 inositol requiring enzyme 1, XBP1 X-box binding protein 1, ERAD ER-associated degradation, bZip basic transcription of leucine zipper, GADD34 growth arrest and DNA damage-inducible 34, ERO-1α endoplasmic reticulum oxidoreductase 1 alpha

The UPR is initiated and regulated by three ER sensors: inositol-requiring enzyme 1 (IRE1), double-stranded RNA-activated protein kinase R (PKR)-like ER kinase (PERK), and activating transcription factor 6 (ATF6). Owing to the binding of BiP, these sensors remain inactive (Fig. 2). The unfolded protein is considered to compete with the BiP binding receptor and results in the activation of three sensors during BiP dissociation, which triggers the UPR.^9^ Typical target genes of UPR can be correlated with protein folding, ERAD, oxidative stress, autophagy, mitochondrial dysfunction, and metabolic pathways that are induced differently due to tissue differences.^10,11^

### Unfolded protein response pathway

#### PERK-eIF2α-C/EBP-homologous protein

The unfolded protein binds to PERK and causes conformational changes, meaning PERK multimerizes and phosphorylates itself.^12^ Then, eukaryotic translation initiation factor 2α (eIF2α), a ubiquitous translation initiation factor, is inactivated by phosphorylation under the activation of PERK, alleviating translation, reducing protein synthesis, and contributing to protein load reduction.^13^ If ER stress persists, ATF4 mRNA translation activates the C/EBP-homologous protein (CHOP) promoter, which controls the target gene expression.^6^

#### IRE1-XBP1

IRE1 is a single-spanning transmembrane protein with dual protein kinase and ribonuclease activities.^14,15^ Once IRE1 is activated, it dimerizes and/or becomes oligomerized, leading to trans-phosphorylation of positive regulatory sites within the protein kinase domain (IRE1 becomes IRE1p), whose changes require adenosine nucleotides (ATP/ADP) as cofactors to exhibit nuclease activity.^14–16^ After activation of the nuclease character of IRE1, it excises an intron (a 26-nucleotide segment) from mRNA encoding a UPR-specific transcription factor called XBP1 (X-box binding protein 1) in metazoans, which transforms the unspliced XBP1 (XBP1u) to the spliced XBP1 (XBP1s).

#### ATF6-ATF6f-bZip

ATF6 is a 90-kDa protein constitutively expressed in cells and is a single-pass type 2 transmembrane protein with a large ER-luminal domain.^17^ It has a cytosolic NH2-terminal domain, which can act as a transcription factor of the basic-leucine-zipper (bZip) family.^18^ Site-1 and Site-2 proteases cleave ATF6 under ER stress. After cleavage, ATF6 releases a cytosolic fragment (ATF6f) that directly controls the transcription of XBP.^19^

### Downstream effect of endoplasmic reticulum stress

ER stress activates all UPR signaling pathways, including protective and pro-apoptosis pathways. However, if the protein level increases before homeostasis restoration, ER stress will be prolonged and the stressed cells will undergo apoptosis.^8,13^ The downstream effects of ER stress can be involved in ERAD, protein synthesis, autophagy, oxidative stress, mitochondrial dysfunction, and metabolism.

#### Protein synthesis and ERAD

ERAD is a part of the ER-mediated protein quality control system, which manipulates the restoration of protein conformation and the clearance of abnormal proteins located on the ER membrane or cytoplasm. The ERAD degradation mechanism can be divided into four steps: substrate recognition by chaperones and lectin, dislocation across the ER membrane driven by VCP/p97, polyubiquitination by E3 ligases, and degradation by the 26S proteasome.^20^ ERAD-L, ERAD-M, and ERAD-C refer to different proteasome degradation substrates of proteins with folding problems or degradation signals, respectively, existing in the ER lumen, transmembrane, or cytoplasmic domain.^21^ ERAD can alleviate ER stress, which can be either induced or inhibited under UPR. Also, prolonged UPR affects protein synthesis, which further aggravates the ERAD deficiency. ER stress can modulate the phosphorylation of eIF2α, leading to the attenuation of protein synthesis, whereas the subsequent activation of ATF4/CHOP can increase protein synthesis, triggering apoptosis.^13^ CHOP encodes a regulatory subunit of an eIF2α-directed phosphatase complex that helps ER-stressed cells recover protein synthesis. Meanwhile, ATF6f released by the ATF6 pathway directly controls the genes encoding ERAD components like Derlin-3.^19,22^ The IRE1/XBP1 pathway is responsible for efficient protein folding, maturation, and degradation in the ER and encodes protein chaperones like ERdj4, p58^IPK^, EDEM, RAMP-4, PDI-P5, and HEDJ.^23^

#### Oxidative stress

Reactive oxygen species (ROS) can be produced in every aerobic cell. Antioxidant systems in cells can significantly prevent ROS production by direct action on radical chain reactions and through detoxifying enzymes like superoxide dismutase (SOD) and catalase, which produce peroxidases.^24^ The generation of ROS relies on several enzymes like nicotinamide adenine dinucleotide phosphate oxidase (NADPH, transforming electrons to molecular oxygen), xanthine oxidoreductase and peroxidases, and mitochondria containing electron transport systems.^24^ When the balance between ROS generation and antioxidant systems is disturbed, oxidative stress (OS) occurs.^25^ ROS links ER stress with oxidative stress. Oxidative stress and ER stress are responsible for cell death resulting from mitochondrial permeability, autophagy impairment, and inflammation. ROS directly activates NF-kB, which facilitates the transcription of inflammation-related cytokines. Growth arrest and DNA damage-inducible 34 (GADD34) is a direct CHOP target gene, that can generate ROS in cells by increasing protein synthesis.^13,26^ ER oxidoreductase (ERO-1α) is vital for disulfide bond formation, which helps proteins fold and transport electrons to molecular oxygen, and facilitates the oxidation of ER proteins. CHOP can upregulate ERO-1α and cause cell apoptosis.^13^ Increased ROS will increase Ca^2+^ to induce apoptosis by activating ITPR3/IP3R (inositol 1,4,5-triphosphate receptor type 3), which is an ER calcium channel.

#### Autophagy

Autophagy can be classified into three types: macroautophagy, microautophagy, and chaperone-mediated autophagy.^27^ Autophagy here mainly refers to macroautophagy. UPR regulates and interacts with autophagy through adenosine monophosphate-activated protein kinase (AMPK), Akt1-MTOR, and MAPK8 transduction.^28^ In particular, mitochondria (mitophagy) and ER (reticulophagy) are involved in ER stress. Under ER stress, an enlarged ER membrane contributes to autophagosome formation. To avoid protein accumulation, ATF4 will activate reticulophagy by facilitating the interaction between ER surface proteins like CCPG1 and ATF8.^29,30^ In reticulophagy, the DDRGK-dependent UFMylation process of ER surface proteins is suppressed by upstream ER stress.^31^ In aging diseases, removing impaired mitochondria by mitophagy in time is vital for cell survival. The PINK2/Parkin pathway involved in mitophagy can be inhibited by eIF2α/ATF4 knockout (KO).^32^ Under ER stress, the eIF2α/ATF4 pathway is essential for autophagy gene transcription, including p62, Nbr1, Atg7, Atg10, Gabarap, and Atg5.^33^ Mammalian oligomerized IRE1 not only cleaves XBP1 mRNA but also activates the stress-induced Jun N-terminal kinase (JNK) through inhibition of autophagy, which interacts with caspase 12.^7^ Inhibiting autophagy can facilitate IRE1 binding to tumor necrosis factor (TNF) receptor-associated factor 2 (TRAF2), which stabilizes its conformation and then interacts with apoptosis signal-regulating kinase 1 (ASK1).^34^ This indicates the IRE1-ASK1-JNK axis is activated in a pro-apoptosis process. Autophagy induced in ER stress can be toxic. Under prolonged ER stress, three branches of UPR are activated, which leads to cell death via a complex consisting of pro-caspase-8 and fas-associating protein with a novel death domain (FADD). This kind of apoptosis is independent of mitochondria and relies on ATG5, which means the involvement of autophagy.^35^

#### Mitochondria dysfunction

Mitochondria dysfunction can manifest as mitochondrial fusion, mitochondrial membrane permeability, transition, pore, and dynamic changes, which will result in NOD-like receptor protein 3 (NLRP3) inflammasome activation, intrinsic apoptosis, oxidative stress, and ER stress. Evidence indicates that the consequences of ER stress can be associated with mitochondrial fusion. The ER and mitochondria are adjacent, and they maintain lipid and Ca^2+^ homeostasis together. The sites where the ER membrane contacts the mitochondrial membrane are called mitochondria-associated ER membranes (MAMs).^36^ Any ER or mitochondrial disturbance can affect the other and initiate a cell response. Under ER stress, IP3R opening leads to the active Ca^2+^ transition between the ER and mitochondria, which facilitates NLRP3 inflammasome activation.^37^ As aforementioned, the Ca^2+^ released from mitochondria results in ER stress. This indicates that MAMs act as the bridge between NLRP3-induced inflammation and ER stress. Mitofusin 2 (Mfn2) is an upstream molecule that suppresses PERK activation and is the bond between UPR and mitochondrial metabolism.^38^ In melanoma, XBP1 facilitates the ubiquitination and degradation of Mfn2, which attributes to mitochondrial fission and mitophagy under ER stress.^39^ Activated CHOP immensely decreases Bcl2, in which BH4-Tat can alleviate the mitochondria membrane potential under ER stress, increases pro-apoptotic protein Bim, and activates caspases like caspase-9, -2, and -3.^40,41^ Subsequently, mitochondrial outer membrane permeabilization facilitates the release of cytochrome c.^35^ Bcl2 can also regulate BH3-only protein expressions (like BAX and BAK), which can bind to mitochondria and cause mitochondrial permeabilization. Therefore, CHOP regulates mitochondrial dysfunction and mitochondria-related intrinsic apoptosis via Bcl2, Bim, and caspases.

#### Metabolism

ER can act as not only the protein quality-control organelle but also the organelle for sterol and phospholipid synthesis and glucose metabolism.^42^ In particular, the cleavage process of ATF6 resembles that of the sterol response element binding protein (SREBP), which is involved in lipid metabolism.^43^ In liver cells, cleaved ATF6 binds to SREBP to form a complex and recruits HDAC1 to downregulate the transcription activity of SREBP.^44^ ATF6 is involved in fatty acid oxidation by interacting with PPARα.^45^ Choline cytidylyltransferase is the limited enzyme in the CDP-choline pathway and can be activated by XBP1s, which presents the lipid biosynthesis induced by IRE1/XBP1.^46^ Furthermore, IRE1/XBP1 regulates normal fatty acid synthesis and β-oxidation by indirectly activating PPARα.^47,48^ PERK/eIF2α regulates glucose and lipid metabolism through C/EBPβ and C/EBPα which directly regulate glucose production and PPARγ.^49^

## ER stress and glaucoma

**Fig. 3:**
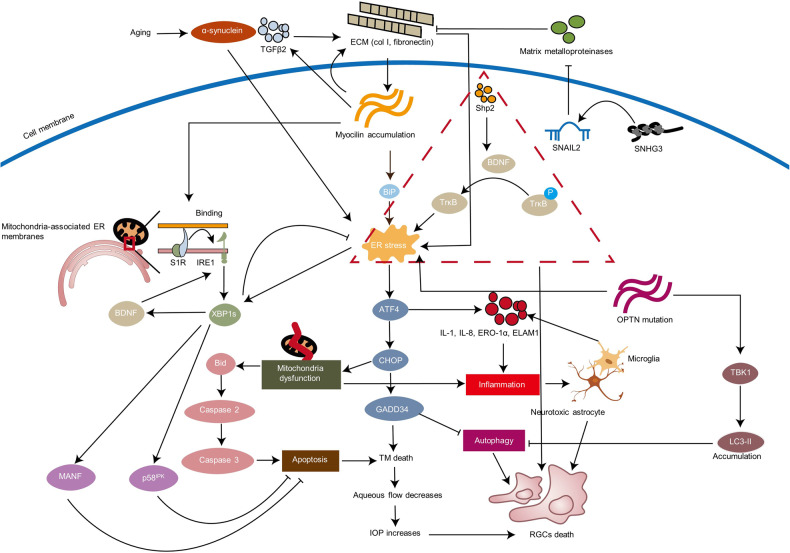
Involvement of ER stress in POAG. POAG can induce aging, aging-related tau, and α-synuclein. Increased ECM, increased TGFβ2, and the accumulation of mutant myocilin can cumulatively lead to ER stress in the trabecular meshwork. Shp2 contributes to ER stress and RGCs loss through the BDNF/TrkB pathway. Following ER stress, the ATF4/CHOP/GADD34 pathway can lead to TM cell apoptosis via Bid/caspase 2/caspase 3, inhibiting autophagy, and stimulating the production of cytokine factors IL-1, IL-8, ERO-1α, and ELAM1. Epigenic modifications like SNHG3/SNAIL2 are involved in the regulation of ECM degradation. OPTN mutation can lead to the accumulation of LCII, which damages autophagy, resulting in ER stress, which induces RGCs death directly. Moreover, mutant OPTN can interact with myocilin accumulation and affect ER stress in TM cells as well. Also, OPTN mutation will facilitate the gliosis which induces cell loss via inflammation. The figure was created with BioRender.com (https://www.biorender.com/). Neurotrophins including P58^IPK^, MANF and BDNF are involved in the protective effect of ER stress. ECM extracellular matrix, BDNF brain-derived neurotrophic factor, MANF mesencephalic astrocyte-derived neurotrophic factor, RGC retinal ganglion cell, POAG primary open-angle glaucoma, TrkB tropomyosin receptor kinase B, OPTN optineurin, SNHG3 small nucleolar RNA host gene 3, SNAIL2 snail family transcription repressor 2

Glaucoma is a heterogeneous group of diseases characterized by cupping of the optic nerve head and visual-field damage, which may result in irreversible blindness and is the second leading cause of irreversible blindness worldwide.^50–52^ A study using UN World Population Prospects data estimated that by 2040, 3.54% of affected people will be 40–80 years old and 111.8 million will be affected overall.^53^ Glaucoma can be classified into three types:^52^ primary glaucoma, which can be divided into open-angle and angle-closure glaucoma, secondary glaucoma, which may result from trauma, certain medications such as corticosteroids, inflammation, tumors, or conditions such as pigment dispersion or pseudoexfoliation, and congenital glaucoma.^54^ Optic nerve damage is common in patients with glaucoma, which is the main cause of vision loss, while trabecular malformations can exist in the pathology of some types of glaucoma, such as primary open-angle glaucoma (POAG). It has been revealed that different risk factors in glaucoma such as aging, glucocorticoid, ischemic, and harmful mutations, will result in chronic UPR, which can be a conserved characteristic in glaucoma and cause the pathological damage mentioned above. We next discuss the role of ER stress in the development of glaucoma and potential targeted treatment (Fig. 3).

### Involvement of ER stress in primary open-angle glaucoma

Although the pathogenesis of POAG is not fully understood, experts have reached a consensus that high intraocular pressure (IOP) is strongly related to retinal ganglion cells (RGCs) death. The increased IOP results in lamina cribrosa transformation and squeezes the optic nerve head, leading to RGCs death involving ER stress. Trabecular meshwork (TM) cells are an essential part of the maintenance of TM function and normal IOP. TM dysfunction is an important pathogenetic factor of POAG in which ER stress plays a crucial role, and the manifestations include a decreased number of TM cells, stiffness of the TM tissue caused by the correlation of the actin reticulum in TM cells, a conformational change of TM beams, and excess accumulation of extracellular matrix (ECM) (including type I collagen and fibronectin). These changes increase aqueous outflow resistance, causing high IOP, RGCs death, and irreversible vision loss. Meanwhile, a subtype of POAG has normal IOP, and the pathology mainly focuses on RGCs death, which involves ER stress. Therefore, we discuss ER stress involvement in affecting TM cell function, ECM remodeling, and RGCs survival in POAG.

#### Function of ER stress in TM death

Gene mutations have a close relationship with chronic ER stress in POAG, leading to TM cell death (Table 1). The most common POAG mutations reside within myocilin (MYOC), which is a gene located on chromosome 1 (GLC1A) that encodes the protein myocilin, including N450Y, Y437H, G364V, Q368X, K423E, I477N, and P370L.^55,56^ Studies have revealed that the MYOC mutation causes about 4% of POAG cases, of which the most common type is juvenile open-angle glaucoma. Disrupting the conformation and production of myocilin by MYOC mutation facilitates mutant myocilin accumulation in TM cells instead of being secreted.^57,58^ In addition, Ca^2+^ imbalance in TM cells acts with the myocilin olfactomedin domain, which facilitates the remaining wild-type (WT) myocilin misfolding and accumulating.^59^ The myocilin accumulation in TM cells and its latter amyloidosis, acting as a key trigger of ER stress in POAG development, induces programmed cell death like apoptosis and impaired autophagy.^60,61^

**Table 1:** The mutations of ocular diseases related to ER stress

Disease	Mutation	Locus and variants	Mechanisms	Reference
Glaucoma	MYOC	N450Y	Trabecular meshwork cell dysfunction	^151,465^
Glaucoma	MYOC	Q368X	Trabecular meshwork cell dysfunction	^466^
Glaucoma	MYOC	Y437H	Trabecular meshwork cell dysfunction	^152^
Glaucoma	MYOC	K423E	Trabecular meshwork cell dysfunction	^467^
Glaucoma	MYOC	I477N	Trabecular meshwork cell dysfunction	^467^
Glaucoma	MYOC	C245Y	Trabecular meshwork cell dysfunction	^468^
Glaucoma	MYOC	D384N	Trabecular meshwork cell dysfunction	^151^
Glaucoma	OPTN	R545Q	Retinal ganglion cell loss	^87^
Glaucoma	OPTN	E50K	Retinal ganglion cell loss	^87^
Glaucoma	OPTN	M98K	Retinal ganglion cell loss	^87^
Glaucoma	TBK1	691_692insAG	Retinal ganglion cell loss	^100^
Glaucoma	WDR36	L25P	Retinal ganglion cell loss	^89^
Glaucoma	WDR36	R529Q	Retinal ganglion cell loss	^89^
Diabetic retinopathy	ALR	C106C	Blood–retinal barrier breakdown	^197,198^
Diabetic retinopathy	ALR	C106T	Blood–retinal barrier breakdown	^197,198^
Diabetic retinopathy	TCF7L2	rs7903146	Neovascularization	^218^
Diabetic retinopathy	IGF1	rs6218	Neovascularization	^222,223^
Diabetic retinopathy	IGF1	rs35767	Neovascularization	^222,223^
Diabetic retinopathy	IGF1	rs35767	Neovascularization	^222,223^
Diabetic retinopathy	VEGF	rs699946	Neovascularization	^224^
Diabetic retinopathy	VEGF	rs833068	Neovascularization	^224^
Diabetic retinopathy	VEGF	rs3025021	Neovascularization	^224^
Diabetic retinopathy	VEGF	rs10434	Neovascularization	^224^
Diabetic retinopathy	TGFβ1	R25P	Neovascularization	^224^
Age-related macular degeneration	HTRA1	rs11200638	Neovascularization	^272^
Age-related macular degeneration	Abca4	D2177N	RPE and photoreceptor death	^279^
Age-related macular degeneration	Abca4	G1961E	RPE and photoreceptor death	^279^
Age-related macular degeneration	Abca4	R1898H	RPE and photoreceptor death	^279^
Age-related macular degeneration	OSBP2	E81E	RPE loss	^281^
Age-related macular degeneration	OSBP2	A150S	RPE loss	^281^
Age-related macular degeneration	OSBP2	S784S	RPE loss	^281^
Age-related macular degeneration	CXCL3	V249I	RPE loss, photoreceptor death, and neovascularization	^282^
Age-related macular degeneration	CXCL3	T280M	RPE loss, photoreceptor death, and neovascularization	^282^
Retinitis pigmentosa	RHO	P23	Photoreceptor cell death	^304–306^
Retinitis pigmentosa	RHO	R135w	Photoreceptor cell death	^306^
Retinitis pigmentosa	RHO	T17M	Photoreceptor cell death	^304–306^
Retinitis pigmentosa	RHO	P53R	Photoreceptor cell death	^304–306^
Retinitis pigmentosa	RH1	G69D	Photoreceptor cell death	^305^
Retinitis pigmentosa	ISBP	D1080N	Photoreceptor cell death	^309^
Retinitis pigmentosa	PDE6	Exon 13	Rod and cone cell death	^314^
Retinitis pigmentosa	PRPF31	A216P	RPE cell death	^322^
Retinitis pigmentosa	PRPF3	S479T	RPE cell death	^322^
Retinitis pigmentosa	PRPF3	T494M	RPE cell death	^322^
Retinitis pigmentosa	PRPF3	A673V	RPE cell death	^322^
Retinitis pigmentosa	PRPF8	R1935H	RPE cell death	^322^
Retinitis pigmentosa	PRPF8	R1935L	RPE cell death	^322^
Retinitis pigmentosa	PRPF8	T1931A	RPE cell death	^322^
Retinitis pigmentosa	IMPG2	Y254C	Cone and rod cell loss	^469^
Achromatopsia	ATF6	Y567N	Cone photoreceptor loss	^350^
Achromatopsia	ATF6	R324C	Cone photoreceptor loss	^350^
Achromatopsia	ATF6	V371Sfs*3	Cone photoreceptor loss	^350^
Achromatopsia	ATF6	Arg324Cys	Cone photoreceptor loss	^346^
Achromatopsia	ATF6	Exons 2, 3, 8–14	Cone photoreceptor loss	^345^
Achromatopsia	ATF6	R376*	Cone photoreceptor loss	^350^
Achromatopsia	CNGA3	ACHM2	Cone photoreceptor loss	^343^
Achromatopsia	CNGB3	ACHM3	Cone photoreceptor loss	^343^
Achromatopsia	PDE6H	10p24	Cone photoreceptor loss	^343^
Achromatopsia	PDE6C	12p13	Cone photoreceptor loss	^343^
Cataract	WFS1	p.Glu809Lys	Len’s opacity	^470^
Cataract	WFS1	c.2425G>A	Len’s opacity	^470^
Cataract	WFS1	p.Glu830Ala	Len’s opacity	^470^
Cataract	WFS1	p.Glu830Ala	Len’s opacity	^470^
Cataract	EPHA2	c.2819C>T	Len’s opacity	^360,361^
Cataract	EPHA2	c.2915_2916delTG	Len’s opacity	^360,361^
Cataract	EPHA2	rs7543472	Len’s opacity	^360,361^
Cataract	EPHA2	rs11260867	Len’s opacity	^360,361^
Cataract	Cx46	p.L11S	Damage of junction and circulation in lens	^471^
Cataract	Cx50	p.P88S	Damage of junction and circulation in lens	^471^
Macular corneal dystrophy	CHST6	Deletion of ORF	Corneal	^420^
Macular corneal dystrophy	CHST6	A1213G	Irregular position of glycosaminoglycan in the stroma	^420^
Macular corneal dystrophy	CHST6	C1301A	Irregular position of glycosaminoglycan in the stroma	^420^
Macular corneal dystrophy	CHST6	G1512A	Irregular position of glycosaminoglycan in the stroma	^420^
Macular corneal dystrophy	CHST6	C840A	Irregular position of glycosaminoglycan in the stroma	^420^
Macular corneal dystrophy	CHST6	replacement of 5′ region	Irregular position of glycosaminoglycan in the stroma	^420^
Macular corneal dystrophy	CHST6	deletion of 5′ region	Irregular position of glycosaminoglycan in the stroma	^420^
Macular corneal dystrophy	CHST6	C.463-464del	Irregular position of glycosaminoglycan in the stroma	^421^
Macular corneal dystrophy	CHST6	C.250_272del	Irregular position of glycosaminoglycan in the stroma	^421^
Granular corneal dystrophy	TGFBI	R124H	Fibroblast dysfunction	^424^
Congenital stromal corneal dystrophy	Decorin	941 (delC)	Fibrillogenesis in extracellular matrix	^422^
Congenital stromal corneal dystrophy	Decorin	967 (delT)	Fibrillogenesis in extracellular matrix	^422^
Schnyder corneal dystrophy	UBIAD1	N102S	Metabolism changes in corneal dystrophy	^430^
Congenital hereditary endothelial dystrophy	Slc4a11	c.743G>A	Endothelilum dysfunction	^416^
Congenital hereditary endothelial dystrophy	Slc4a11	c.1033A>T	Endothelilum dysfunction	^416^
Fuchs endothelial corneal dystrophy	COL8A2	Q455K	Endothelium dysfunction and EXM accumulation	^428^
Keratoconus	SOD1	c.169+50 delTAAACAG	Fibrocell and endothelium dysfunction	^441^
Uveitis	NOD2	R334W	Uvea inflammation	^385^
Uveitis	NOD2	R334Q	Uvea inflammation	^385^
Uveitis	NOD2	E383K	Uvea inflammation	^385^
Uveitis	NOD2	G481D	Uvea inflammation	^385^
Uveitis	NOD2	W490S	Uvea inflammation	^385^
Uveitis	NOD2	M513T	Uvea inflammation	^385^
Uveitis	NOD2	R587C	Uvea inflammation	^385^
Uveitis	NOD2	N670K	Uvea inflammation	^385^
Uveitis	Calpain	R243L	Uvea inflammation	^387^
Uveal melanoma	GNAQ	Q209L	Melanocyte disturbance	^397^
Uveal melanoma	EIF1AX	Not really known	Melanocyte proliferation	^401^

ATF4 acts as the most significant upstream regulator of ER stress in TM cells.^62^ First, it aggravates the myocilin accumulation in TM cells. ATF4 directly acts with misfolded proteins and WT myocilin, which results in reduced secretion of myocilin and aggravates the cytotoxic function in TM cells. Excess ER stress facilitates defects in ERAD, which worsen myocilin accumulation.^63^ The presence of GRP94 induced by ER stress helps mutant myocilin escape from ERAD, and since mutant myocilin exists as an amyloidogenic protein, stimulating ubiquitin-proteasome degradation is limited.^61^ Then, ER stress in TM cells induces damage, including oxidative stress, inflammation, impaired autophagy, and mitochondrial dysfunction, and results in a morphological change of human TM cells and disruption of the normal cell cycle, which affects normal TM function. ER stress leads to oxidative stress and inflammation via activation of the ATF4-CHOP-GADD34/ERO-1α pathway, presenting inflammatory cytokines such as interleukin (IL)-1, IL-4, ROS, IL-8, endothelial leukocyte adhesion molecule 1, and cleaved caspase-3.^62,64,65^ Mitochondrial swelling can be found under mild ER stress during TM cell death.^66^ The ATF4-CHOP pathway activates impaired autophagy as the downregulated autophagy flux facilitates TM cell death.^67,68^ Inhibition of mTOR with rapamycin significantly saves TM cells, indicating the harmful function of autophagy in TM cells.^55^ As discussed above, the resulting TM cell dysfunction will affect the normal ECM. TM cells phagocytose debris from functional TM tissues to keep aqueous humor flowing. TM cells dynamically synthesize and degrade ECM, which provides an architecture to TM tissues and a normal humor aqueous outflow pathway. The overexpression of ATF4 impairs the phagocytic activity of TM cells which contributes to ECM accumulation.^62^ Also, ECM accumulation (fibronectin and actin) can lead to ER stress in TM cells.^58,69–71^ Transforming growth factor β2 (TGFβ2) increases in the aqueous humor of MYOC^Y437H^ mutation patients and facilitates ECM accumulation, which activates ER stress.^70–74^ In conclusion, protein aggregation in TM tissues, the proliferation of ECM, and changes in cytokines such as TGFβ2 dynamically interact to regulate IOP. Therefore, MYOC mutation leads to ER stress, which affects the lifespan of the TM cells, and ECM remodeling, resulting in glaucoma.^60,75–78^

Epigenetic modification is essential for correct myocilin folding and refers to stable and heritable changes in gene expression or cellular phenotype without changes in Watson–Crick DNA base-pairing.^79^ The core regulates chromatin structure and gene expression by covalently modifying histone proteins and nucleic acids, including DNA methylation, histone modifications, RNA modifications, non-coding RNA, and chromatin remodeling.^80^ Under OS, long non-coding RNA (lncRNA), small nucleolar RNA host gene 3 (SNHG3), and the snail family transcription repressor 2 (SNAI2) are upregulated in human TM cell culture, in which SNHG3 binds to ELAVL2 to stabilize SNAIL2 mRNA.^81^ Matrix metalloproteinases (MMPs) are a family of metzincin proteases that cleave the components of the ECM in TM and modulate TM architecture. SNAI2 decreases MMP3 and MMP9 activity, which destroys the balance of synthesis and ECM degradation and increases the aqueous humor outflow resistance.^82^ SNAI2 activation is dependent on the XBP1 pathway in carcinoma cancer.^83^ It indicates that SNAI2 in TM cells is responsible for TM cell death, and ECM accumulation is dependent on XBP1 and is under the control of ER stress. Thus, lncRNA and ER stress participate together to facilitate ECM accumulation and TM dysfunction.

N-linked glycosylation, a post-translational modification (PTM), is involved in myocilin synthesis and processing and attaches oligosaccharides to asparagine residues in the N-terminal domain of the protein.^67,68^ The glycosylated myocilin proteins will form disulfide bonds to achieve the correct spatial conformation. Therefore, inhibition of PTM results in protein accumulation and ER stress in TM cells, which causes TM cell loss and ECM accumulation. Tunicamycin, a broadly used ER stress inducer, acts by inhibiting N-linked glycosylation.

#### Function of ER stress in RGCs loss

Gene mutations in POAG are significant causes of RGCs loss, such as those in Optineurin (OPTN), TBK1, and WDR36, which are associated with ER stress in RGCs (Table 1). OPTN is an adapter protein mainly expressed in the cytoplasm and the autophagy receptor of the cell,^76,84^ whose mutation results in autophagy dysfunction, impaired signal transduction, and protein accumulation.^85,86^ OPTN mutations including E50K, R545Q, M98K, and 691_692insAG can be found in POAG with high IOP and normal IOP.^87^ TBK1 is associated with the autosomal dominant inheritance of normal glaucoma. Duplications and triplications of TBK1 are related to normal IOP glaucoma.^88^ The interaction of TBK1 and OPTN determines that the pathology of these two gene mutations is inextricably linked. WDR36 is widely expressed in eye tissues, such as the retina and optic nerve. Although the precise effect of the WDR36 mutation in glaucoma remains unknown, it can result in protein dysfunction and may lead to ER stress.^89^

OPTN mutation causes optic nerve axon degeneration and then RGCs loss through ER stress. As the optic disk rim loses nerve tissue, the lamina cribrosa recedes and oppresses the optic nerve.^90^ Neuronal axon transportation is important for neuronal function and RGCs survival, as RGCs deliver nutrients through axons to synapses, and aged organelles or signaling vesicles are retrogradely transported to the soma.^91^ The crushing and destruction of the optic nerve can recruit microglia and astrocytes, a process called gliosis. Mitochondrial fragments and inflammation cytokines like IL-1α, TNF-α, and C1q released by microglia facilitate the transition of astrocytes to a neurotoxic reactive type, which results in RGCs death.^92–94^ CHOP and BiP are localized in GFAP-positive astrocytes with the OPTN^E50K^ mutation, indicating ER stress involvement in the activation of neurotoxic reactive astrocytes and RGCs loss.^95^ The morphology of mitochondria changes before the axon in aged OPTN^E50K^ mice, and RGCs degeneration and OPTN mutated cells fail to initiate mitochondria autophagy.^96^ MAM disturbance activates ER stress and induces RGCs apoptosis.^97^ OPTN mutations can also affect the RGCs soma through ER stress. RGCs with OPTN mutations tend to possess protein accumulation through blocked autophagy and interaction with TANK-binding kinase 1 (Tbk1). The WT OPTN protein facilitates the transportation of ubiquitin or ubiquitinated aggregates to the autophagosome, whose mutation will cause large molecular protein degradation dysfunction and aggregates formation. TAR DNA-binding protein 43, which is responsible for mRNA processing and trafficking and microRNA biogenesis clearance, is blocked in RGCs, resulting in neurodegeneration like amyotrophic lateral sclerosis (ALS).^98^ TAR DNA-binding protein 43 accumulation has been proven to be closely related to ER stress and neuronal death.^99^ OPTN^691_692*ins*AG^ mutation can interact with Tbk1, which leads to LC3-II protein accumulation and directly contributes to cell death via ER stress.^100^ Furthermore, OPTN^E50K^ enhances the affinity for Tbk1 and increases the insoluble OPTN protein in RGCs.^86^ As aforementioned, abnormal protein accumulation will induce ER stress, and persistent accumulation will cause RGCs damage. Besides the direct activation of ER stress, OPTN^M98K^ makes RGCs more susceptible to damage caused by ER stress than WT RGCs and enhances apoptosis.^101^ The RGCs energy metabolism balance is disturbed by the OPTN^E50K^ mutation. Impaired autophagy in OPTN-mutated mice, as aforementioned, will compensatively downregulate mTOR1. AMPK in RGCs, an energy homeostasis regulator and stress sensor, is activated via the downregulation of mTOR1, which is a pathway responsible for neurite growth and stem cell proliferation.^102^

In other models without gene mutations, ER stress also plays a crucial role in RGCs loss. In a DBA/2J mouse model of chronic glaucoma, neurofilaments in the optic neuronal nerve were lost, and the ER stress marker CHOP was colocalized with the neuronal nerve.^103^ ER stress decreases expression of Mfn-1 and Ace-tubulin, which contributes to mitochondrial fusion in spinal cord-injured neuron axons. Axon degeneration might further induce mitochondrial dysfunction in RGCs. The mitochondria fusion and rupture increase B-cell lymphoma 2 (Bcl-2) and decrease Bcl2-associated X (BAX) expression, which induces apoptosis. Besides classic intrinsic apoptosis induced by mitochondrial dysfunction, this change can induce a unique Bid-caspase2-caspase3 pathway to induce RGCs death.^104^ Aging is a risk factor for POAG. CHOP increases with age, and XBP1 decreases in human eye tissue.^105,106^ In the aged human retina, protein aggregates of non-phosphorylated tau and α-synuclein increase substantially, further supporting the presence of protein misfolding and the resulting ER stress.^107^ In a micro-bead-injected mouse model and a silicon oil-induced ocular hypertension mouse model, the high IOP and RGCs loss are accompanied by CHOP elevation.^108^

CHOP KO in vivo or XBP1s overexpression contributes to RGC survival, indicating a complicated and dual function of ER stress in RGCs.^109^ Protein accumulation in RGCs contributes to the binding of the Sigma-1 receptor (S1R) to IRE1.^110,111^ The S1R resides on the MAM, described as the “pluripotent modulator,” which only chaperones the ER stress sensor IRE1 to facilitate inter-organelle signaling for survival.^112^ It can transiently stabilize IRE1 by binding to it. Consequently, the period of conformational IRE1 is prolonged, and the downstream activity of splicing XBP1 increases, which can lead to cell survival.^112^ Furthermore, under OS, S1R prefers to bind to and phosphorylate BiP, which reduces its ability to refold protein.^113^ Therefore, XBP1s are upregulated modestly and transiently, whereas CHOP increases dramatically during the injury process. The total effect of ER stress is detrimental to RGCs survival.

The interaction between ER stress and neurotrophic factors plays an important role in glaucoma pathogenesis. p58^IPK^ is an ER-resident chaperone, playing a critical role in facilitating protein folding and protein homeostasis, which protects RGCs from damage like apoptosis under ER stress.^23,114,115^ Meanwhile, the induction of p58^IPK^ depends on XBP1.^23^ The mesencephalic astrocyte-derived neurotrophic factor is a member of a newly identified ER-localized neurotrophic factor family and is upregulated during ER stress.^116^ Upregulated mesencephalic astrocyte-derived neurotrophic factor can protect RGCs from hypoxia-induced apoptosis by inhibiting CHOP.^117^ p58^IPK^ and mesencephalic astrocyte-derived neurotrophic factor can act together, which provides a protective function for RGC restoration.^114^ Brain-derived neurotrophic factor (BDNF) facilitates neuron regeneration, whose transcription relies on XBP1 splicing.^118^ Activated BDNF will form a positive loop for the IRE1-XBP1 pathway.^119^ Also, BDNF can prevent the upregulation of CHOP in RGCs and reduce RGCs loss.^120^ Src homology region 2-containing protein tyrosine phosphatase 2 is related to IL-1-induced Ca^2+^ signaling.^121^ Its overexpression can dephosphorylate the TrkB receptor, and BDNF/TrkB neuroprotective survival signaling is reduced, resulting in ER stress and RGCs apoptosis.^122^ Nerve growth factor (NGF) belongs to the neurotrophic factor family and is essential for mature and immature neural cells. In glaucoma, decreased NGF facilitates RGCs apoptosis because NGF ameliorates the expression of the IRE1-JNK-CHOP signaling pathway and reduces Bcl2 and Bad.^123^ The mechanisms underlying neurotrophic factors and ER stress are still unknown.

### Involvement of ER stress in other types of glaucoma

#### ER stress and acute glaucoma

ER stress, which modulates inflammation and immune response, is involved in acute glaucoma. IRE1α is demonstrated to facilitate translocation of NF-κB to release inflammation cytokines such as IL-6, IL-7, and TNF-α, which results in whole retina inflammation, especially RGCs death.^124^ Also, IRE1α can induce ROS, which then facilitates NLRP3 binding to mitochondria and activates caspase-2 to lead to mitochondria-related apoptosis.^125^ In acute glaucoma models, NLRP3 is involved in RGCs pyroptosis, apoptosis, necrosis, ferroptosis and PANoptosis, indicating the potential role of ER stress in mediating RGCs loss through NLRP3 activation.^126–130^ In acute glaucoma mouse models, the CXC-motif chemokine ligand 10/CXC-motif chemokine receptor 3 (CXCL10/CXCR3) axis is activated via ER stress, which can promote microglial recruitment, inflammation, and mediate leukocytes.^131–133^ The activated CXCL10/CXCR3 axis causes thinning of the retinal ganglion layer, indicating the role of ER stress as the regulator of the retinal immune axis.

Epigenetic modulations, including histone acetylation and methylation, are involved in ER stress and RGCs loss in acute glaucoma. Mice under ischemic/reperfusion (IR) injury present acute IOP elevation and optic neuron injury, which can mimic acute glaucoma. Histone deacetylase (HDAC) 6, an enzyme that deacetylates lysine residues on histones or other proteins in the cytoplasm and nucleus, is upregulated in IR models.^134^ BiP expression is under the control of the acetylation of histone H3 and histone H4 Arg3 methylation. YY1 recruits P300 (responsible for acetylation) and PRMT1 (responsible for methylation) to the BiP promoter to enhance BiP expression.^135^ BiP can bind to caspase 12 and block CHOP activation-induced apoptosis. Under IR injury, HDAC elevation decreases BiP expression, which upregulates CHOP and leads to RGC death.^136^

PTM participates in RGC loss via ER stress as well.^137^ Peroxiredoxins (Prxs) are a family of peroxidases that can reduce peroxide by oxidizing a specific region of a conserved cysteine residue.^138^ Acetylation of Prxs will increase their antioxidant ability. HDAC6 targeting Prx2 is specifically increased in glaucoma, which reduces the defensive ability of RGCs under stress.^139^ In neurons, disruption of Prx4 causes ROS to increase and subsequently induces ER stress.^140^ Thus, HDAC induces ER stress by deacetylating Prxs and causing RGCs loss.

#### ER stress and glucocorticoid-induced glaucoma

Glucocorticoids (GCs) are the most widely used medication worldwide. However, persistent use results in secondary glaucoma. Evidence of the involvement of ER stress in GC-induced glaucoma shows a higher level of BiP, GRP94, and CHOP and more phosphorylation level of IRE1α and eIF2α.^141^ Exerting GCs will result in the overload of MYOC levels and ECM proteins in TM cells, which can induce UPR, cause TM dysfunction, and elevate IOP.^69,141^ TGFβ2 signaling plays an essential role in glucocorticoid-induced ocular hypertension.^142^ Under ER stress induced by glucocorticoid, the TGFβ2/SMAD3 pathway is activated, which facilitates ECM deposition and actin accumulation and induces ER stress.^141,142^ Therefore, glaucomatous characteristics like elevated IOP and RGCs death can result from the simultaneous function of ER stress and TGFβ2.

#### ER stress and pseudoexfoliation

Pseudoexfoliation (PEX) syndrome is a late-onset disease characterized by the deposition of fibers and ECM accumulation.^143^ Statistical data reveals that 25% of people with PEX develop glaucoma, called PEX glaucoma which possesses an imbalance in ECM accumulation and subsequent activation of ER stress as BiP and PERK are upregulated.^144^ In addition, SYVN1, an E3-ubiquitin ligase, is downregulated, indicating decreased proteasome activity, which disturbs protein homeostasis and enhances ER stress in the lens capsule of PEX glaucoma.^145^ Then, caspase-3, caspase-12, and CHOP levels increase, indicating apoptosis and downstream retinal ER stress.

### Therapeutic targets for glaucoma

Currently, the only effective method to treat glaucoma is to lower the intraocular pressure.^146^ The main treatment goals are slowing disease progression and preserving the quality of life.^54^ Many medicines aim to reduce intraocular pressure, including prostaglandin analogs, β-adrenergic blockers, α-adrenergic agonists, carbonic anhydrase inhibitors, and cholinergic agonists.^147^ In particular, the drugs used in POAG are effective only in a few glaucoma cases. Therefore, new drugs aimed at ER stress are required.

#### Treatment targeting ER stress for repairing damaged TM in glaucoma

ER stress is involved in TM cell dysfunction and loss. Currently, there are chemicals, natural compounds, gene therapies, and stem cell therapies aimed at ER stress.

Grp94 is responsible for protein homeostasis and degradation. In MYOC mutant TM cells, Grp94 recognizes myocilin olfactomedin and facilitates myocilin accumulation, which induces ER stress and TM cell dysfunction or loss.^148^ Inhibition of Grp94 facilitates mutant myocilin degradation through autophagy rather than ERAD at first and decreases accumulation.^63^ 4-Br-BnIm was proved to be safe and can selectively inhibit Grp94, which facilitates mutant or misfolded myocilin degradation through effective autophagy in TM cells.^149^ PERK, the upstream component of the UPR branch, can initiate apoptosis and DNA damage in TM cells, which affects the cell cycle, morphology, and function. LDN-0060609, a PERK inhibitor, exists in aqueous solution as ketone, enol, and enolate, and can save TM cells from ER stress.^150^ 4-Phenylbutyric acid (4-PBA), an aromatic short-chain fatty acid, can enhance the outflow of mutant myocilin.^63,151^ 4-PBA can also degrade the ECM by activating MMP9.^152^ Furthermore, in GC-induced glaucoma, 4-PBA alleviates ER stress, like CHOP expression in the TM tissue, and decreases IOP.^141^ Astragaloside-IV, once used in renal and cardiac diseases, is effective for preventing myocilin deposition.^153^ Astragaloside-IV can rescue TGF-β2 induced ocular hypertension by modulating ECM deposition and ER stress in the TM by interacting with the MMP3 and MMP9 systems.^153^ Regarding the specific MYOC^D384N^ mutation resulting in POAG, trimethylamine N-oxide, a natural osmolyte, can act as an ER chaperone, which alleviates myocilin misfolding and rescues TM cells from ER stress-induced apoptosis.^151^

Considering the importance of MYOC mutations and ER stress in the pathogenesis of POAG, many researchers have developed new therapies targeting mutant genes. Clustered regularly interspaced short palindromic repeats (CRISPRs) and Cas proteins are broadly expressed in bacteria and archaea.^154^ The CRISPR-Cas9 system, which is an immune system using RNA-guided nucleases to cleave foreign genetic elements, is commonly used in gene editing. By introducing a single-guide RNA into the CRISPR coding region to achieve specific recognition, target genes can be identified through complementary base pairing. The Cas9 protein can recognize the protospacer-adjacent motif sequence located upstream of target genes.^155^ Subsequently, cas9 promotes gene editing by inducing DNA double-strand breaks. This technology can be used to insert, replace, and delete target genes by introducing different single-guide RNAs. MYOC mutation results in protein accumulation and later ER stress-induced TM damage and ECM remodeling. MYOC^Y437H^ KO by CRISPR-Cas9 assembly Ad5-crMYOC alleviates myocilin accumulation and ER stress, restoring IOP and vision function.^77^ RNA interference can precisely modulate gene expression in mammalian cells. Small interfering RNA (siRNA) is a component of the RNA interference complex that silences specific genes with complementary sequences.^156^ After siRNA formation via Dicer or direct introduction into cells, it will form an RNA-induced silencing complex. Subsequently, siRNA binds to the target mRNA through complementary sequences, while an argonaute protein, like endoribonuclease, in the RNA-induced silencing complex cleaves target mRNA to achieve the silence of target genes.^157^ The introduction of siRNA to reduce mutant MYOC expression contributes to the repopulation of TM cells, prevention of RGCs loss, and recovery of IOP via inhibition of ER stress.^75^ As ECM remodeling in TM tissue has a close relationship with the overexpression of fibronectin, CRISPR-Cas9 targeting fibronectin successfully downregulates the ECM deposition and reduces ER stress, which protects TM function.^69^

Stem cells are self-renewable and able to differentiate directionally from functional cells, which can be used in the treatment of a variety of diseases. Human trabecular meshwork stem cells (TMSCs) are extracted from human TM tissue and can differentiate into adipose cells, neuronal cells, and TM cells.^158^ The pluripotent stem cell marker OCT4 and the neural stem cell marker Nestin can be detected in TMSCs.^158,159^ Similar to mesenchymal stem cells (MSCs) with directional homing characteristics, TMSCs injected into the anterior chamber can orient homing to TM through the interaction of highly expressed CXCR4 receptors on TMSCs with the SDF1 molecule in TM tissue.^160^ The highly expressed integrin, α5β1, on the TMSC membrane promotes the anchorage and survival of homing TMSCs in TM tissue by interacting with fibronectin, an ECM component of TM tissue.^161^ Considering the homing and differentiation functions, transplantation of TMSCs to treat MYOC mutations resulting in TM cell dysfunction is viable. TMSC transplantation can facilitate aqueous humor outflow, recover normal IOP, rescue RGCs function, and reduce RGCs death.^162^ The potent mechanism is that TMSCs homing TM differentiates into TM cells with phagocytic function, which facilitates relief of ER stress, replication of endogenous TM cells, and the restoration of ECM structure.^162^ Also, TMSCs are less sensitive and can survive in strong ER stress environments.^66^ These results indicate the possibility of stem cell therapy by transplantation of TMSCs in the future. Induced pluripotent stem cells (iPSCs) and MSCs can be induced into cells resembling TM cells in vitro.^163^ MSCs can be distributed to TM and reduce RGCs loss.^164^ Combining stem cell therapy with superparamagnetic iron oxide nanoparticles is proven to be safe for MSCs and helps stem cells anchor and function in TM tissue accurately by using magnetic field positioning.^165^ Adipose-derived stem cells can integrate into TM tissue and normalize IOP, whose homing ability is directed by CXCR4/SDF1, which vividly reduces ER stress induced by mutant TM cells.^166^

Exosomes are thought to be the main mediators of MSCs paracrine effects. MSCs-derived exosomes have low immunogenicity and are relatively safer than stem cell therapy. Direct treatment with MSCs exosomes can achieve a similar therapeutic effect as stem cell transplantation, meaning stem-derived exosomes have become a “cell-free therapy” option for a variety of diseases. Exosomes derived from bone marrow MSCs protect TM cells from oxidative stress and inflammation. The non‐pigmented ciliary epithelium can secrete exosomes rich in miRNA and cytokines, which function on the TM to regulate cell proliferation and ECM. Treating TM cells with NPCE-derived exosomes causes decreased COL3A1 expression. The miR29b component in the exosome is proved to affect autophagy and downregulate molecules involved in ER stress, including CHOP, ATF6, eIF2α, and XBP1.^167^ miR29b can also downregulate the WNT/β‐catenin pathway, responsible for ECM production.^168^ In addition, nrf2 enriched in non‐pigmented ciliary epithelium-derived exosomes can protect TM cells from oxidative stress, which can directly inhibit the apoptosis pathway and indirectly reduce TM cell loss by alleviating ER stress.^169^

#### Treatment targeting ER stress for rescuing RGCs in glaucoma

RGCs loss is a common reason for vision loss, in which ER stress plays an essential role. Treatments targeting ER stress include chemicals, neurotrophic factors, approved drugs in other fields, and gene therapy.

In acute glaucoma, ischemia resulting in hypoxia and malnutrition causes RGCs to lack ATP, inducing ER stress. Thus, RGCs undergoing ER stress in acute glaucoma are subject to cell death. ATPase Kyoto University Substances (KUSs) are inhibitors of valosin-containing protein (VCP) ATPase, which can be exerted by intravitreal injection.^170^ KUSs can alleviate CHOP induction by modulating key genes, such as Zfp667, and have been shown to save vision in rats by protecting RGCs, amacrine cells, and photoreceptors.^171,172^ The PERK-ATF4-CHOP pathway is involved in RGCs death and retinal axon degeneration via apoptosis and other programmed cell death under stress, while the IRE1α-XBP1 pathway is activated in RGCs and protective for RGCs survival. Directly mediating UPR molecules can be a novel strategy for glaucoma. Using adeno-associated viruses (AAV) to deliver proteins and mediate the UPR pathway can be protective for RGCs. Combining CHOP or eIF2α KO by CRISPR/Cas9 with XBP1 activation by AAV-XBP1s provides a synergistic function, that saves both the neuron axon and RGCs soma, and restores vision function.^173^ AAV-mediated Grp78 injected into the retina can be transported to RGCs and reduces apoptosis by attenuating ER stress through downregulating CHOP, ATF4, and eIF2α.^174^ KO of CHOP or ATF4 alone by CRISPR/Cas9 relieves UPR downstream DNA damage and facilitates neuron axon regeneration.^175^ Histamine receptor H1-mediated Ca^2+^ release and ER stress in RGCs can be blocked by amoxapine, desloratadine, and maprotiline.^108^ These drugs can inhibit all three UPR pathways and protect RGCs, and they have been approved by the Food and Drug Administration (FDA) to be safe in clinical trials. This protective effect can also be achieved by HR1H deletion in RGCs using CRISPR/Cas9 technology.^108^ siRNA aimed at ER stress has been verified to protect RGCs. RNA-dependent PKR phosphorylation facilitates eIF2α activation, which leads to RGCs loss. Thus, inhibition of PKR by an imidazolo-oxindole derivative or siRNA can relieve ER stress like CHOP reduction and promote RGCs survival.^176^ Valdecoxib, a selective COX2 inhibitor that has been used to treat osteoarthritis and arthritis, can reduce RGCs apoptosis in vivo and in vitro via inhibiting ER stress activation like the PERK-ATF4-CHOP pathway.^177^ p58^IPK^ overexpressed by AAV can elevate RGCs survival rates through refolding proteins and inhibition of ER stress.^115^ Also, p58^IPK^ might prevent mitochondria-related cell death and provide cells with increased expression of neurotrophins like BDNF. Nervous excitability toxicity and high IOP are correlated with ER stress-induced RGCs death in glaucoma models. Retinoid X receptors, originally highly expressed in ganglion cell layers, are downregulated in glaucoma retinas and induce ER stress. Elevation of retinoid X receptor expression in the retina by resveratrol can reduce ER stress-induced apoptosis and mitochondrial dysfunction.^178^ It can also repress HDAC1 in RGCs. As aforementioned, HDAC activity is associated with ER stress gene transcription, such as CHOP and BiP, indicating HADC inhibition can be a novel target for inhibition of ER stress in RGCs. Tubacin, an HDAC6 inhibitor, restores prx2 expression, which is a retinal antioxidant, and reduces retinal degeneration.^134^ Valproate, a bipolar disorder and epilepsy drug, can inhibit HDAC as well and is proven to increase the expression of BiP and decrease CHOP, which disturbs harmful ER stress and protects RGCs.^136^ Drugs targeting ER stress and autophagy to rescue RGCs with OPTN mutations have been studied. Rapamycin could restore the impaired autophagy in RGCs with OPTN mutations and recover RGCs function. While rapamycin inhibits the mTOR pathway, which is harmful to RGCs proliferation, a new drug, trehalose, can induce autophagy in OPTN^E50K^ retinal organoids independent of the mTOR pathway which decreases OPTN spot accumulation and normal neuron morphology and function compared with WT RGCs.^102^ Considering the close relationship between ER stress and OS, combining drugs with nanoparticles to deliver inhibitors at the same time can be more effective. A glaucomatous microenvironment-responsive degradable polymer has been designed, characterized by thioketal bonds and a 1,4-dithiane unit in the main chain as well as pendant cholesterol molecules. Thioketal bonds and the 1,4-dithiane unit are responsible for erasing ROS in RGCs while cholesterols are used to target the cell membrane.^179^ Designing nanoparticle-wrapped drugs to target ER stress could provide innovative therapy.

As aforementioned, S1R can be released and upregulated in glaucoma, which can prolong the protective signal pathway of IRE1-XBP1, while the protective effect of S1R is limited under stress. SR1 ligand (+)-pentazocine can prevent the inhibition effect of S1R on BiP and reduce the phosphorylation of BiP, which reduces ER stress activation as PERK, ATF4, ATF6, IRE1α, and CHOP are downregulated, and saves RGCs in vitro.^113^ Neurotrophic factors regulate neural system development and function. Utilizing neurotrophins can directly support neuron regeneration, restoring their function.^180^ Furthermore, considering the reduced content of neurotrophic factors, which are correlated with ER stress, applying BDNF and mesencephalic astrocyte derived neurotrophic factor (MANF) can inhibit the upregulation of CHOP and prevent RGCs apoptosis.^117,120^ Also, recombinant human NGF prevents RGCs loss and was shown to be safe and effective in a phase 1b randomized controlled study of POAG patients.^181^

## ER stress and diabetic retinopathy

Diabetic retinopathy (DR) is the most common and serious ocular complication of diabetes mellitus.^182^ DR is the leading cause of vision loss in developed countries.^183^ The number of diabetes patients worldwide reached 410 million in 2015 and is estimated to reach 640 million in 2040. About 40% of type 2 diabetes patients and 86% of type 1 diabetes patients have diabetes retinopathy. Diabetes retinopathy is classified as non-proliferative or proliferative. Non-proliferative diabetes retinopathy is characterized by the destruction of the blood–retinal barrier (BRB) function. It causes retinal edema, hemorrhage, and exudation. Proliferative diabetes retinopathy manifests as pathological retinal angiogenesis (fibrovascular membrane formation along with the vitreoretinal interface, vitreous hemorrhage, and retinal detachment). Retinal microvascular disease in diabetic patients is often accompanied by retinal neurodegeneration, which is an important reason for decreased vision in diabetic patients. It has been shown that different metabolic pathways are involved in the occurrence of diabetes retinopathy and neuropathy, such as an increase in the polyol pathway, advanced glycation end products, and activation of protein kinase C. It can induce ER stress in retinal cells and cause pathological changes in diabetic patients. We focus on the role of ER in the development of diabetic retinopathy and neuropathy, and describe therapy against it.

### Involvement of ER stress in diabetic retinopathy

**Fig. 4:**
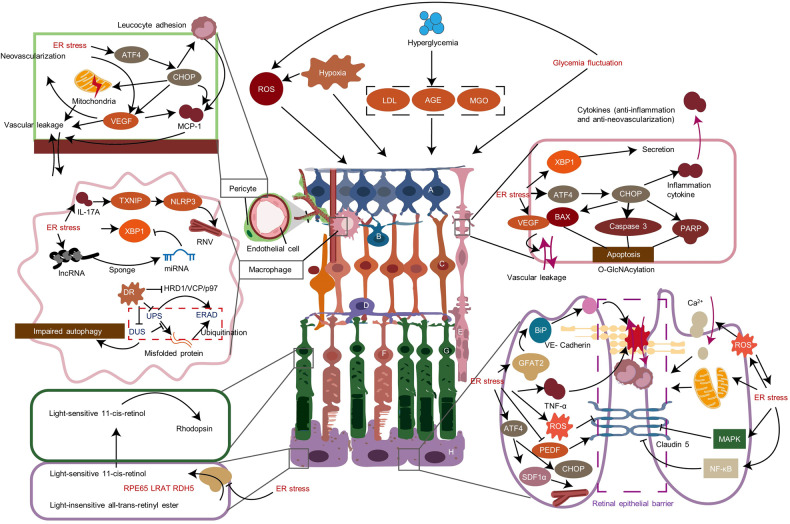
Involvement of ER stress in DR. Many factors can activate ER stress in a DR model, including hyperglycemia, hypoxia, ROS accumulation, products like LDL, AGE and MGO, and glycemia fluctuation. In pericytes, activated ER stress can induce the ATF4/CHOP pathway and then activate mitochondrial dysfunction, VEGF, and MCP-1, which facilitates leukocyte adhesion and vascular leakage. In DR, macrophages accumulate and facilitate RNV through ER stress or the IL-17A/TXNIP/NLRP3 pathway. Following ER stress, XBP1 facilitates protective anti-inflammatory and anti-neovascularization cytokines. In addition, the ATF4/ CHOP pathway to contributes inflammation, RNV production, and apoptosis of BAX, caspase 3, and PARP. The RPE cells are the most important component of the outer epithelial barrier. ER stress injures the barrier by destroying VE-cadherin and Claudin 5 through O-GlcNAcylation of VE-cadherin/Grp78, MAPK pathway, NF-κB activation and inflammation. ER stress affects the barrier directly through ROS production, mitochondrial membrane potential loss, and PEDF decrease. ATF4/SDF1α leads to RNV in RPE. Impaired autophagy and lncRNA are involved in the development of DR as well. The key enzyme of producing light-sensitive 11-*cis*-retinol is suppressed, which influences vision. a. retinal ganglion cell; b. amacrine cell; c. bipolar cell; d. horizontal cell; e. Müller cell; f. cone cell; g. rod cell; h. retinal pigment epithelium (RPE). The figure was created with BioRender.com (https://www.biorender.com/). DR diabetic retinopathy, LDL low density lipoprotein, AGE advanced glycation end product, MGO methylglyoxal, VEGF vascular endothelial growth factor, RNV retinal neovascularization, NLRP3 NOD-like receptor protein 3, PEDF pigment epithelium-derived factor, RPE retinal pigment epithelium

ER stress mainly functions in DR via BRB breakdown, retinal neovascularization, and neuron damage (Fig. 4). Metabolic changes can facilitate DR and ER stress in DR retinas and can provide metabolism sensors for glucose fluctuations (hypoglycemia and elevated glucose), O-GlcNAcylation, low-density lipoprotein, copper, and advanced glycation end products (AGEs).^184–186^ All three UPR pathways are activated in the DR process.^187–194^

#### Function of ER stress in BRB breakdown in DR

BRB can be divided into inner and outer components, with the inner BRB being formed by tight junctions between neighboring retinal capillary endothelial cells and the outer barrier by tight junctions between retinal pigment epithelium (RPE) cells.^195^ Besides, Müller cells and pericytes are important for the maintenance of normal inner BRB function. Since ER stress is activated in cells composing the BRB in DR, the importance of BRB destruction in the pathogenesis of DR becomes apparent.^192,196^

Various candidate gene association studies have revealed an immense association between genetic factors and the development of BRB damage, including the involvement of aldose reductase, which led to the accumulation of sorbitol through the polyol pathway (Table 1).^197,198^ Aldose reductase contributes to reduced NAD^+^ levels and inhibits sirtuin protein, leading to an increase in protein acetylation, which may be correlated with O-GlcNAcylation. Also, aldose reductase attributes to Müller glia (MG) activation. Aldose reductase C(-106)T polymorphism is a DR risk.^198^

PTM and epigenetic modulation, including glycosylation and the lncRNA/miRNA axis, are also involved in BRB damage. Glycosylation of plasma membrane and secretory proteins is a global phenomenon called post-translational modification (PTM).^199^ The glycosylation process can occur in the cytosol, ER, or Golgi complex. Glycosidases and glycosyltransferases are the essential processors of glycosylation.^200^ O-GlcNAcylation is a key PTM based on the addition of a single monosaccharide, β-O-D-N-acetylglucosamine (β-O-GlcNAc), ontoserine, or threonine residues of nuclear, cytoplasmic, and mitochondrial proteins.^201^ ER stress plays an important role in the O-GlcNAcylation of endothelial junction protein, resulting in inner BRB breakdown.^202^ During high glucose conditions, O-GlcNAcylation is enhanced by upregulating the expression of glutamine-fructose-6-phosphate aminotransferase 2 (GFAT2), which increases the flux from the hexosamine biosynthesis pathway, leading to the formation of uridine 5′-diphosphate-*N*-acetylglucosamine, the substrate for PTM, loss of retinal endothelial barrier integrity, and transendothelial migration of monocytes.^192^ Also, under treatment of ER stress inducers in retinal endothelia cells, translocation of GRP78 to the plasma membrane increases O-GlcNAcylation of proteins, including focal adhesion kinase, a known regulator for vascular permeability; cathepsin D, which is responsible for endothelial permeability; and particularly VE-cadherin and β-catenin, which result in defective complex partnering.^202^ A high-fat diet in mice has been shown to contribute to ER stress in MG and increase expression of O-GlcNAcylation protein.^192^ ER stress (especially eukaryotic translation in initiation factor 4E, eIF4E) activates the lipotoxicity sensor nuclear receptor subfamily 4 group A member 1 (NR4A1) and GFAT2 to induce O-GlcNAcylation protein.^192,203^ The O-GlcNAcylation phenomenon in MG might destruct the BRB by upregulating CD40 expression and increasing inflammation.^203^ The fluctuation of ncRNAs caused by hyperglycemia exists in RPE cells, and their function can be harmful or beneficial for different ncRNAs.^188,189^ ER stress is associated with ncRNAs in the pathogenetic processing of outer BRB breakdown. lncRNA growth arrest‑specific transcript 5 has multiple cell functions, including promotion of vascular endothelial growth factor (VEGF)-A, apoptosis (decreasing Bcl/BAX ratio), and pyroptosis.^204^ In ARPE cells, hyperglycemia downregulates expression of growth arrest‑specific transcript 5, which inhibits ER stress by interacting with sarcoplasmic/ER Ca^2+^ ATPase 2, which leads to inflammation and BRB injury.^188,205^ miR-204 directly targets and downregulates sirtuin-1 lysine deacetylase in RPE. The activation of the miR-204/sirtuin 1 axis worsens ER stress and leads to apoptosis, presenting as an elevation of cleaved caspase-3, -9, -12, and poly ADP-ribose polymerase.^189^

ER stress and OS influence each other regarding BRB damage and result in cell death through inflammation, apoptosis, and tight junction protein degradation. Under hyperglycemia, the ATF4/CHOP pathway is activated, and apoptotic-related molecules increase in MG, including BAX, cleaved caspase-3, and poly ADP-ribose polymerase, through the ATF4/CHOP pathway.^187,206^ One of the mechanisms of ER stress-induced MG apoptosis is that glyceraldehyde 3-phosphate dehydrogenase is transported and localized in the nucleus by binding Siah-1 under triglyceride treatment.^207^ RPE cells undergoing OS and ER stress have a decreased change in tight junction protein ZO-1 and occlusion.^208^ Under mild ER stress and chronic proinflammation, a feed-forward loop is formed in the endothelial junction protein, in which TNF-α is exacerbated and visual deficits are caused in the retina.^209^ Also, following the mitogen-activated protein kinase (MAPK) pathway and NF-κB activation, claudin5 is downregulated among tight junction proteins.^210,211^ Furthermore, intermittent high glucose may result in the activation of the ATF4-CHOP pathway, which can facilitate the release of MCP-1 in pericytes.

Mitochondrial dysfunction correlates with the metabolic pathway, apoptosis, and inflammation in the pathology of BRB destruction. Stimulator-of-interferon genes (STING) are vital for sensing cytosolic DNA and initiating innate immune responses against microbial infection and tumors and are located in the ER as homodimers. It can be activated by cyclic guanosine monophosphate (GMP)-adenosine monophosphate produced by the key DNA sensor cyclic GMP-adenosine monophosphate synthase and transported to Golgi binding TBK1 and interferon regulatory factor 3. The cGAS-STING axis also activates the NF-κB pathway, resulting in IFN production and inflammation activation, which releases cytokines like IL-1β, TNF-α, and IL-6.^212^ In human retinal vascular endothelial cells, the IRE1α and ATF6 pathways are upregulated under hyperlipidemia, which facilitates mitochondrial dysfunction and results in mitochondrial DNA leakage.^213^ As the most abundant and potent STING ligand, mitochondrial DNA stimulates STING activation and enhances the immune response, causing microvascular hyperpermeability.^213^ ER and mitochondrial coupling is accompanied by elevated mitochondrial calcium ions (Ca^2+^) and mitochondrial dysfunction in AGE cultured endothelial cells and apoptosis^214^ via IP3R1-GRP75-VDAC1 which can be inhibited by 4-PBA. In human retinal pericyte death, mitochondria dysfunction is characterized by mitochondrial membrane potential loss and cytokine c release.^184,215^ The RPE is an important constituent of the outer retinal barrier, and its damage equals the damage to the retina. Accumulation of AGEs and their adduct, methylglyoxal, in RPE can produce ROS, which activates mitochondrial membrane potential loss, intracellular calcium elevation, and an ER stress response to induce RPE cell death.^216^ Overexpression of mfn2 to induce mitochondrial merging also contributes to RPE death.^186^ Key isomerases like RPE65, LRAT, and RDH5 that convert light-insensitive all-trans-retinyl ester to light-sensitive 11-cis-retinol for continued visual function in RPE decrease under ER stress.^194^

The dual function of autophagy in human retinal pericyte survival has been researched. When treated with low-dose low-density lipoprotein, increased autophagy markers like beclin-1 and LC-3 facilitate cell survival, while impaired and exaggerated autophagy is induced under severe oxidative and ER stress. JNK phosphorylation is essential to autophagy induced by low-density lipoprotein and ER stress, which implicates PERK–eIF2a and IRE1–JNK signaling pathways in autophagy. Unlike JNK, which is involved in apoptosis in other cells, it is CHOP that accelerates pericyte apoptosis.^184,215^

#### Function of ER stress in retinal neovascularization in diabetic retinopathy

Neovascularization is one of the most important pathogenetic changes in DR, and different cytokines involved in the neovascularization process and the disturbance of normal cell function are related to ER stress.

There is ample evidence that gene polymorphisms play a prominent role in the pathogenesis of DR via ER stress in neovascularization. Gene variation in transcription factor 7 like 2 (TCF7L2) is a susceptible contributor to PDR (Table 1).^217^ The T allele of TCF7L2 rs7903146 is associated with fibrovascular membrane formation in type 2 diabetes mellitus-PDR patients.^218^ As part of the Wnt pathway, TCF7L2-regulated pathological neovascularization and VEGFA generation occur in diabetic models via ER stress-dependent pathways.^218,219^ The distribution of TCF7L2 is mainly in the cell nucleus of the RGCs layer and the inner nuclear layer. TCF7L2 overexpression in retinal progenitor cells (RPCs) affects endothelial cell transcription and leads to microvascular permeability.^217^ Increased ER stress markers like eIF2α indicate that ER stress elevates the microvascular generation function of rs7903146, and the T risk variation confers additional susceptibility to ER stress. VEGF can induce vascular permeability and retinal neovascularization, ultimately leading to microvascular damage and retinal dysfunction.^220^ In a survey conducted in a Han Chinese population, insulin-like growth factor 1 gene polymorphisms (rs6218, rs35767, and rs35767) were associated with DR.^221^ Insulin-like growth factor 1 facilitates the MG to induce neovascularization, whose expression can be upregulated under ER stress.^222,223^ The single-nucleotide polymorphisms of VEGF, including rs699946, rs833068, rs3025021, and rs10434, have an immense correlation with blinding DR in T1DM and type 2 diabetes mellitus.^224^ ER stress facilitates VEGF secretion, indicating the correlation between single-nucleotide polymorphisms of VEGF and ER stress in DR pathology. TGFβ1, the encoding protein, can be facilitated via ER stress activation and is involved in the TGF-β/Smad3 signaling pathway in regulating glucose and energy homeostasis.^225,226^

Epigenetics is defined as “the study of changes in gene function that are mitotically and/or meiotically heritable and that do not entail a change in DNA sequence.”^227,228^ Epigenetic changes involve both DNA and chromatin molecular modifications that change the expression of genes and genome activity and include DNA methylation of CpG dinucleotide residues, histone modification, most ncRNAs, RNA methylation, and chromatin structure.^228^ In the last decade, epigenetic modulations have been broadly studied in type 2 diabetes mellitus, but few conclusions have been drawn regarding DR.^229^ lncRNA metastasis-associated lung adenocarcinoma transcript-1 is found to contribute to inflammation and epigenetic regulation in DR^205^ and suppresses Grp78 production to regulate ER stress and alleviate inflammation and angiogenesis.^205^ It also sponges and inhibits miR-125b expression, which can suppress retinal neovascularization characterized by VE-cadherin and VEGF downregulation.^230^

High glucose results in antioxidant elimination involving SOD, which involves eliminating superoxide radicals; CAT converts harmful H_2_O_2_ into H_2_O and O_2_, while GR regulates physiological glutathione levels to stabilize ROS levels.^231^ OS and ROS release can be the early changes in DR and act as upstream regulators of ER stress, and all three UPR branches are involved.^232^ In particular, ATF4 suppresses antioxidant enzymes to increase ROS and induce OS. Also, ATF4 stabilizes HIF1α and plays a crucial role in generating VEGF. In addition, ER stress is the causal factor for inflammation and angiogenesis, coupled with VEGFA upregulation, overexpressed inflammatory cytokine transcription like TNF-α, IL-6, NF-κB, IL-17A, and ICAM-1,^233,234^ and inflammation cell infiltration. For example, the proinflammatory cytokine IL-17A is involved in macrophage polarization, which can induce M1 macrophage polarization. In response to hypoxia, the interplay between IL-17A and ER stress contributes to retinal neovascularization via modulation of the TXNIP/NLRP3 signaling pathway.^234^ TPL2 (tumor progression locus 2) is downstream of proinflammatory cytokines. Sensing AGEs, the TPL2/SDF1α (stromal cell-derived factor-α) axis, which regulates vascular generation, is activated in the RPE, resulting in microvascular dysfunction.^235^ Therefore, OS, ER stress, and inflammation are correlated and function as a cascade to generate cell death and VEGF production. Notably, retinal neovascularization can trigger retinal inflammation, which forms a loop between ROS, ER stress, inflammation, and VEGF. MG activation is found to be one of the main factors in DR onset and progression.^236^ Regarding infection or OS (like diabetes), MG is activated and regulates pro-angiogenic factors like pigment epithelium-derived factor (PEDF) and VEGF dependent on the activation of the UPR pathways.^237–239^

#### Function of ER stress in neuron damage in diabetic retinopathy

When exposed to pathogenic factors of DR, ER stress in the retina is correlated with retinal degeneration through inflammation, apoptosis, and autophagy.

Müller cell abnormalities, including gliosis, activated proliferative and migrative activities, inflammation cytokine release, and dysregulation of neuronal guidance cues, are key events in DR pathogenesis and can result in neuron damage. Semaphorins constitute a large family of endogenous secreted and transmembrane-associated proteins. The role of the secreted protein Sema3A (collapsin-1) is to induce the contraction and collapse of structures on axon growth cones.^240^ Under high glucose in the early stage, ER stress induces MG secreting Sema3A and is protective for MG resistance to overactivation of ER stress via inhibiting the IRE1α/XBP1 pathway.^240^ However, in the late stage, Sema3A’s protective function was inadequate, and neuronal degeneration occurred. Inflammation cytokine infiltration in neurons evokes IRE1α, and PERK-eIF2α-ATF4 pathways are involved in DR neurons, which in turn correlates with the generation of inflammatory factors IL-6 and MCP-1.^188,190^ Meanwhile, RGCs are lost via ER stress-induced apoptosis.^241^

ER-mitochondria cross-talk plays a vital role in neuronal death. PERK inhibits translation by adding a phosphate group to eIF2α, which stimulates the expression of TXNIP by activating transcription factors ChREBP and ATF5. As a result, TXNIP inhibits the ROS scavenging activity of TRX1 through a disulfide exchange reaction between the redox domains of TRX. Uncombined TXNIP will bind to TRX2 in mitochondria, which can act as key regulators of the NLRP3 inflammasome. This process facilitates mitophagy and dynamin-related protein 1 (Drp1-SNO) interaction with mitochondrial fission 1 protein to trigger the mitochondrial fission process.^242^

The ubiquitin–proteasome system is responsible for cleaning misfolded and non-functional proteins and is correlated with ERAD through the binding of HRD1 (an E3 ligase) and VCP/p97 (an ATP-driven chaperone governing the ubiquitin–proteasome system).^243^ Downregulation of deubiquitination enzymes, LCB3 and VCP/p97 in DR illustrates an impaired ubiquitin–proteasome system and low ERAD efficiency, resulting in neuron damage.^187^

### Therapeutic targets for diabetic retinopathy

#### Treatment targeting ER stress for BRB damage in diabetic retinopathy

ER stress plays an important role in the destruction of the BRB. At present, a variety of natural extracts, chemicals, and gene therapies are used to treat diabetes retinal microvascular disease by alleviating ER stress. Natural chemicals are relatively safe, and attractive study options. Astragalus polysaccharide is a traditional Chinese Medicine and a bioactive polysaccharide extracted from *Astragalus membranaceus* root (Huang qi), which has been broadly employed clinically for its antitumor and antidiabetic properties.^244,245^ ER stress is involved in outer BRB damage and the occurrence of macular edema in patients with diabetes. It is associated with the induction of programmed death in RPE cells and the destruction of tight junctions between the cells. It has been demonstrated that astragalus polysaccharide can repair the destroyed diabetic outer BRB by alleviating inflammation and reducing the apoptosis of RPE cells through the miR-204/sirtuin 1 axis.^189^ Lactucaxanthin is a xanthophyll carotenoid predominantly presenting in lettuce that functions as an antioxidant and antidiabetic substance with anticancer properties dependent on tissues.^246–248^ It can directly reach the retina region and relieve symptoms via ER stress inhibition through potent antioxidant activity by downregulating PC, malonyl dialdehyde, inflammatory markers, OS inhibition, and HIFα induced VEGF reduction.^208,233^ Furthermore, it can decrease vascular leakage by rescuing the destroyed tight junctions between RPE cells in diabetic models.^208^ Chrysin is a flavone-type flavonoid that exists in honey, propolis, honeycomb, and passion flowers and exhibits multiple biological effects, including anti-inflammation and neuroprotection. Chrysin can reduce ER stress in RPE cells. It can reverse aberrant production of VEGF, insulin-like growth factor-1, and PEDF in glucose-incubated RPE cells, which contributes to restoring impaired BRB. Furthermore, chrysin could repair the retinoid visual cycle by alleviating ER stress via AGE-RAGE activation in diabetic models.^194^ Elevated copper levels have been found in the serum of patients with DR. Copper synergistically interacts with high glucose to induce ER stress and inflammation in RPE cells through modulation of mitochondrial function and changing the expression of the mitochondrial fusion protein 2. It eventually causes outer BRB dysfunction. Copper chelation with penicillamine can relieve copper-induced toxicity in RPE. It can reverse outer BRB dysfunction through reduced ER stress and ameliorate mitochondrial fusion protein 2-associated mitochondrial dysfunction in RPE cells under diabetic conditions.^186^

ER stress is associated with the loss of retinal vascular endothelial cells, pericytes, and Müller cells, which contributes to diabetic inner BRB breakdown. Tauroursodeoxycholic acid (TUDCA) protects cells from DR damage by inhibiting GRP78 translocation, VE-cadherin O-GlcNAcylation, ER-induced apoptosis, and reducing VEGF generation and vascular leakage.^204^ TUDCA could reverse ER stress-induced damage to vascular endothelial cells via Takeda G protein-coupled receptor 5, suggesting TUDCA is a potential therapeutic candidate for diabetic inner blood barrier damage.^249^ Nobiletin, a polymethoxylated flavone extracted from citrus explants, can be transported from the blood to retinal tissue. ER stress induces GADPH nuclear translocation, which causes Müller cell death and inner BRB destruction. Meanwhile, the death of Müller cells results in decreased PEDF levels and an imbalanced VEGF/PEDF ratio, which exacerbates inner BRB damage. Nobiletin has been proven to protect Müller cells from HG-induced apoptosis by relieving ER stress in the cells, which facilitates the repair of diabetic-induced iBRB disruption and rebalances the VEGF/PEDF ratio.^207^ Ghrelin, a gastric-derived acylated peptide, regulates energy homeostasis by transmitting information about peripheral nutritional status to the brain and mainly binds to the growth hormone secretagogue receptor-1a, a seven-transmembrane G protein-coupled receptor.^250^ It is essential for protecting organisms against famine and is widely distributed in human cells. An advantage of ghrelin is that it can reach ocular tissues through the BRB. Ghrelin protects the inner BRB from high glucose (HG) injury by inhibiting activation of the PERK pathway and reducing ER stress in retinal vascular endothelial cells.^250^ As aforementioned, chronic hyperglycemia and hyperlipidemia are involved in DR. Hyperlipidemia can induce ER stress and activate the STING signaling pathway in retinal vascular endothelial cells, which is associated with inner BRB breakdown. IRE1α, as an ER stress sensor, can be knocked out by CRISPR-Cas9 technology in retinal vascular endothelial cells, which attenuates the STING signaling pathway and reduces mitochondria leakage by inhibiting IRE1α/XBP1 signaling and alleviating ER stress in the cells. It might provide a novel strategy to treat diabetic inner BRB breakdown.^213^ RNA interference has been used to treat diabetic retinal microvascular abnormalities by inhibiting ER stress in retinal cells. The transcriptional factor TCF7L2 participates in diabetic damage of the inner BRB. siRNAs targeting TCF7L2 could suppress ATF6 signaling-mediated ER stress and rescue inner BRB breakdown in vascular endothelial cells under high glucose conditions. A 58-kilodalton inhibitor of protein kinase is a member of the heat shock protein 40 family. It is an initiator of eIF2α phosphorylation, which plays a key role in the PERK-induced UPR response.^251^ siRNA against P58^IPK^ was found to exacerbate diabetic vascular leakage, while overexpression of P58^IPK^ inhibited ER stress-induced CHOP activation and VEGF elevation in retinal vascular endothelial cells.

#### Treatment targeting ER stress for neurodegeneration in diabetic retinopathy

Diabetic retinal neurodegeneration has been found in patients with diabetes mellitus. It causes vision to decrease even in the absence of diabetic microvascular disease. Neurotrophic factors are used to treat diabetic neuropathy. Neurotrophin-4 (NT-4) is a member of the well-known neurotrophin family that regulates neuronal networks by regulating neuronal survival, differentiation, growth, synaptic development and plasticity, and myelination. NT-4 relieves ER stress in DR and reduces RGCs injury, which can be considered a potential drug for diabetic neuron damage.^241^ New technologies have developed a complex of NT-4 with dendrimer nanoparticles.^252^ The NT4-polyamidoamine electrostatic complex can provide a sustained concentration of protein in vitreous and retinal tissues over an extended period after delivery and promote retinal, especially RGCs, recovery from injury.^252^ Chemical drugs that could directly inhibit ER stress have been investigated to protect retinal neuronal cells from diabetic injury. Liraglutide, a glucagon-like peptide-1 analog, is widely used in the clinic and has a protective effect on neurodegenerative diseases. Liraglutide can also treat diabetic neuropathy through activation of the Erk pathway and regulation of the Trx-ASK1 complex. It subsequently inhibits ER stress and OS in retinal neuron cells. 4-phenylbutyric acid (4-PBA) is a chemical chaperone that mimics endogenous chaperone activity to resolve ER dysfunction in pathological conditions. Administration of 4-PBA could decrease retinal neuron death in the outer nuclear layer and ganglion cell layer by attenuating ER stress in these cells in diabetic models.^253^ Sulforaphane is widely discovered in cruciferous plants and is a strong antioxidant and activator of nrf2.^254^ Sulforaphane inhibits ER stress via the AMPK pathway, and it can also reduce inflammation, apoptosis, and OS in retinal cells. It has been demonstrated that sulforaphane can prevent photoreceptor cell death under diabetic stimulus.^254^ A combination of stem cell therapy with chemical drugs provides a synergetic therapeutic effect on retinal neurodegeneration. Melatonin, a neuroendocrine hormone mainly synthesized in the pineal gland, is involved in pleiotropic biological functions, including control of the circadian rhythm, immune enhancement, and antioxidant, anti-aging, and antitumor effects. Melatonin targeting ER stress function has been revealed recently. It mainly functions in neural cells and is neuroprotective, which mainly inhibits CHOP and then PERK and GRP78/BiP.^255^ Combining melatonin and adipose-derived MSCs significantly delays the progression of diabetic neuropathy and retinopathy.^256^

## ER stress and age-related macular degeneration

Age-related macular degeneration (AMD) occurs in the macular region and gradually affects central vision.^257,258^ AMD is the third leading cause of irreversible blindness worldwide, usually affecting people aged >55 years.^259^ AMD was estimated to affect 196 million people in 2020 and is expected to rise to 288 million by 2040, with the largest number of cases in Asia (113 million).^260^ Currently, AMD can be classified into two major categories: dry AMD and neovascular AMD (nAMD). nAMD is characterized by a choroidal neovascularization complex, subretinal or intraretinal fluid, hemorrhage, and/or fibrous scar tissue. Dry AMD manifests as the loss of RPE cells overlying photoreceptors and underlying choroidal capillaries.^258^

Though the concrete pathology of AMD is unclear, it is recognized that physiological and conformational changes happen on photoreceptors, RPE cells, Bruch’s membrane, and the choriocapillaris.^261^ RPE cells possess many functions for retinal homeostasis, including maintaining the outer BRB and secretion of proteins.^262^ Proteins secreted by RPE include PEDF, αB crystallin, and prosaposin, which can suppress angiogenesis and reduce drusen.^263^ First, intracellular debris, including lipofuscin, accumulates in the RPE, which causes RPE injury and ECM accumulation. As the lesion is aggravated, lipid-rich debris is laid down between the basal lamina of the RPE and the inner collagenous layer of Bruch’s membrane, involving RPE debris, lipids, minerals, and immune system-associated elements.^264^

**Fig. 5:**
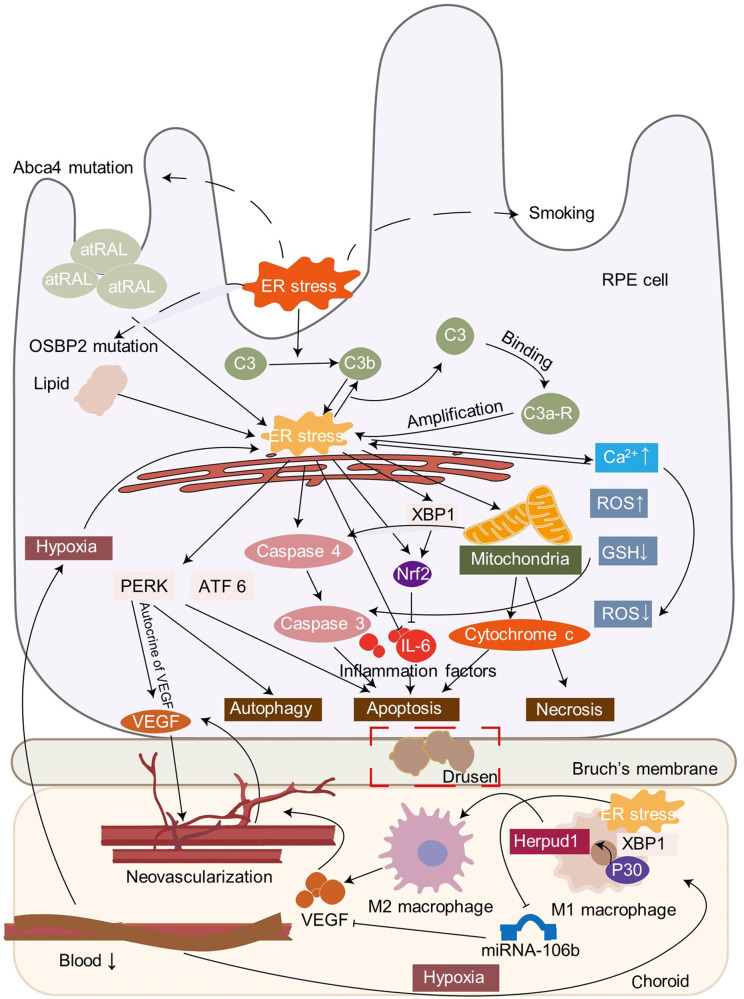
Involvement of ER stress in AMD. Many risk factors contribute to AMD development, including smoke and light. The pathogenesis of AMD is closely associated with RPE death. Complement C3 can be activated and transformed into C3b to induce ER stress. The latter activated ER stress provokes the binding of C3a to C3a receptors, which amplifies the ER stress. ER stress promotes the inflammation in AMD to directly induce inflammatory factors like IL-6 and IL-8. In addition, XBP1 can provoke the protective nrf2 pathway to activate inflammation. Under ER stress, it activates caspase-4, generates ROS, elevates intracellular calcium, and reduces the mitochondrial membrane potential to promote the downstream activation of caspase-3, inducing apoptosis. Mitochondrial damage releases cytochrome c leading to RPE death. Neovascularization and ER stress influence each other through endocrine of VEGF. What’s more, decreased blood in choroid can induce hypoxia and ER stress in RPE cell and macrophages in retina. The polarization of macrophage depends on epigenic modifications of XBP1 gene which facilitates VEGF release and causes neovascularization. The figure was created with BioRender.com (https://www.biorender.com/). VEGF, vascular endothelial growth factor. AMD age-related macular degeneration, nrf2 nuclear factor erythroid2-related factor 2, RPE retinal pigment epithelium, VEGF vascular endothelial growth factor

Risk factors for AMD involve both genetic and environmental factors. Age, race, self-conditions such as basic diseases (diabetes, hypertension, etc.), and complement system activation play important roles in the incidence of AMD.^265^ Smoking is the strongest environmental risk factor for nAMD. Other elements, including diet, sunlight, glucose deprivation, and complements such as C3 and C3a, accelerate AMD progression.^266–268^ These risk factors can induce ER stress in AMD models.^269–271^ Research has revealed that different metabolic pathways, including the cholesterol pathway, retinoid metabolism, and lipid metabolism, are involved in drusen accumulation, neovascularization, and subretinal fibrosis via ER stress. We elaborate on the role of ER stress in the formation of AMD and its targeted treatment (Fig. 5).

### Involvement of ER stress in age-related macular degeneration

#### ER stress and Neovascular age-related macular degeneration

Nowadays, genome-wide association studies identify variants associated with AMD (Table 1). The high-temperature requirement A1 (HTRA1) gene (rs11200638) is positive for anti-VEGF therapy.^272^ HTRA1 belongs to the high-temperature requirement A family and performs dual peptide refolding and degradative chaperone actions, which can be activated to defend against damage under UPR.^273^ HTRA1 disturbance presents a reduced ability to deal with proteotoxicity and cell apoptosis, indicating the correlation between HTRA1 variants and ER stress in the development of nAMD.^273^

ER stress-related epigenetic modifications are a vital part of nAMD pathology. ER stress promotes aberrant choroidal neovascularization (CNV) by modulating the expression of angiogenesis-associated genes. In nAMD models, the activated PERK/eIF2α pathway leads to the downregulation of miRNA-106b in vascular endothelial cells. miRNA-106b directly regulates VEGF and HIF-1α expression by targeting its 3′-UTR in vascular endothelial cells. Decreased miRNA-106b promotes the formation of CNV by increasing the proliferation and migration of vascular endothelial cells.^209^ Reduced blood flow in the choroid and retinal tissue hypoxia is associated with the occurrence of CNV in patients with nAMD. Hypoxia can increase the acetylation and protein stability of XBP1 by enhancing histone acetyltransferase p300, which activates UPR-associated molecules ATF4, GRP94, BiP, and CHOP and triggers UPR in macrophages. Activation of ER stress controls macrophage polarization from M1 to M2, which increases VEGFA. Hence, hypoxia induces p300 activation and regulates UPR and M2 polarization in macrophages, which increases paracrine secretion of VEGFA and promotes the proliferation, migration, and tube formation of choroidal vascular endothelial cells.^274^

OS interacts with ER stress, leading to AMD lesions. A combination of various stresses can be summarized as the integrated stress response (ISR), in which ER stress usually occurs upstream of the ISR and activated ISR.^275,276^ Amyloid-β protein precursor (AβPP) is a constituent of drusen, which can accelerate ER stress and ISR.^277^ ISR facilitates VEGF production and angio-vascularization in endothelial and RPE, in which ATF4 is the main contributor to CNV formation in AMD.^274,275^ Inflammation might be the mediator between VEGF and ER stress via the MAPK signaling pathway and the P300/XBP1s/Herpud1 axis.^278^

#### ER stress and dry age-related macular degeneration

Gene mutations and variants involved in dry AMD include phototransduction, sterol toxicity, and protein-folding dysfunction (Table 1). Abca4, localized on human chromosome 1p21, exists in AMD.^279^ Variants of Abca4 associated with AMD include D2177N, G1961E, and R1898H.^279^ The Abca4 protein is an ATP-binding cassette transporter 4 and is responsible for the elimination of metabolic products from phototransduction or lipids. Its mutation results in incomplete RPE phagocytic function and lipid deposition. As Abca4 carries all-trans-retinal out of the photoreceptor disc, its mutation injures retinoid metabolism by releasing free all-trans-retinal which is toxic for RPE and photoreceptors. The toxicity of all-trans-retinal is produced by direct activation of PERK/eIF2α/ATF4/CHOP signaling, which stimulates downstream apoptosis-inducing molecules like p-JNK, p-c-Jun, cleaved caspase-3, and cleaved-PARP.^280^ The oxysterol-binding protein gene is mainly expressed in the RPE, localized in the macular region, and its protein binds to and transports oxysterols. As the macula ages, oxysterols gradually accumulate and become cytotoxic for RPE, activating ER stress. Nucleotide changes in the oxysterol-binding protein, including c.347A>G, c.450G>T, and c.2351G>A, are found in patients with dry AMD.^281^ Single-nucleotide polymorphisms in CXCL3 are risk variants for AMD.^282^ ERp29 deficiency resulting in protein accumulation in CCL2^−/−^/CX3CR1^−/−^ mouse models might explain the ER stress activation and related AMD.^283^

ER stress regulated by PTM is involved in dry AMD. Choroidal blood vessel atrophy leads to RPE loss through blood and glucose deprivation. In this circumstance, glycosylation of the VEGFA receptor peptide is incomplete.^284^ Simultaneously, a perturbation of normal glycosylation leads to VEGFA receptor accumulation, contributing to excessive ER stress, which acts as the connection between PTM and cell loss.

ISR significantly participates in dry AMD development. The whole retina undergoing ER stress produces ROS. Components of the retina, including MG and RPE, react to ER stress and reduce damage. The MG maintains homeostasis in the internal environment, including oxidative redox status and glutamate homeostasis. In response to ER stress, the MG exerts a protective role by upregulating AβPP to defend against apoptosis and recover functional gene transcription.^269^ Interestingly, AβPP is proven to be harmful in neovascularization AMD, as mentioned before. These data seem to convey that AβPP is a dual regulator of cell survival dependent on VEGF expression level. Under OS disturbance, RPE cells and their tight junctions are damaged, and BRB integrity is damaged. Meanwhile, Erp29, an ER chaperone protein, is upregulated to modulate redox status by increasing nrf2 and inhibiting ER stress in RPE, which enhances BRB integrity.^270^ When undergoing mild ER stress, activation of XBP1 upregulates the nrf2 expression level, which is protective for RPE.^285,286^ However, under severe ER stress, these compensatory responses are not sufficient to protect the cells. In the hydrogen-peroxide-induced cell viability loss of RPE, ferroptosis is the main cause of RPE death, which affords new perspectives for AMD pathology.^287^ Therefore, UPR is protective when the injury is mild but harmful when damage persists.

Since lipid drusen is the most important lesion in AMD, lipid metabolism is involved in ER stress, contributing to dry AMD. Evidence shows that the complement system in combination with ER stress leads to lipid accumulation in the RPE and Bruch’s membrane, in which ER stress is activated by complement C3 and C3a.^266,267^ Activation of ER stress directly stimulates SREBP and disturbs lipid homeostasis.^288^

Mitochondrial dysfunction in RPE has a close relationship with ER stress. Mitochondria are important for RPE integrity.^289^ In a model of AβPP deficiency, ER stress is overexpressed in RPE and results in cell apoptosis through the mitochondrial dysfunction center, which manifests as caspase-4 and caspase-3 upregulation, decreased BCL/BAX ratio, increased release of cytochrome c and glutathione, and mitochondria membrane potential loss.^216,290^ Under abundant ROS production, mitochondrial fission activates mitophagy to eliminate damaged mitochondria. The morphology changes of mitochondria including swelling and breaking is induced by ER stress, and this dysfunction induces RPE necrosis.^291^

Autophagy is excessive and impaired under ER stress.^283^ The normal function of RPE includes phagocytosis and digestion, in which the normal function of autophagy-lysosome-dependent ERAD is crucial to remove accumulated abnormal protein and protein aggregates. PERK-activated autophagy increases over time.^292^ However, severe and persistent ER stress causes prolonged autophagy and autophagy flux through the PERK pathway and even results in RPE self-death.^268^ Thus, light injury ER stress is activated and causes dry AMD-like retinal lesions, including drusen, RPE alteration, and photoreceptor degeneration.^283^ EIF2AK3 downregulation has been discovered in patients with AMD, which lowers the PERK level.

### Therapeutic targets for age-related macular degeneration

Zinc, antioxidants such as ascorbic acid and vitamin E, lutein/zeaxanthin, and omega-3 (docosahexaenoic acid) used alone or in combination can delay AMD progression. Inhibition of VEGF is effective in treating neovascular AMD. For example, pegaptanib, bevacizumab, and aflibercept. However, AMD treatment lacks efficacy.

Natural plant extracts can safely and effectively target ER stress in AMD. Grape polyphenols relieve symptoms in both dry AMD and nAMD.^293^ Curcumin is a yellow pigment found in the rhizome of turmeric. It saves RPE from ER stress, OS, and mitochondrial dysfunction, showing potential for dry AMD treatment.^294^ Chemicals can also be potential treatments in the future. 4-PBA, the classic ER stress inhibitor, can act on both types of AMD. It suppresses choroidal neovascularization by inhibiting VEGF production and can prevent RPE death in a dry AMD model. Many other drugs can be used in dry AMD, such as paeoniflorin^295^ and propofol,^296^ which affect the molecular BAX and apoptosis processes of ER stress. As for nAMD, ISR inhibition can prevent the production of ATF4 and VEGF and act as a treatment target.^275^

These chemicals can be extracted directly from the human body and have been proven to be effective in dry AMD treatment. Humanin is a 21–24 aa peptide encoded by the mitochondrial MT-RNR2 gene encoding 16S rRNA discovered in surviving neurons in patients with Alzheimer’s disease. Humanin protects RPE cells from oxidative stress-induced cell death and affects not only the ER stress-related pathway but also mitochondrial-ER-associated membranes, which facilitate RPE survival in dry AMD.^297^ Taurine is one of the most highly expressed amino acids in the eye tissue. In RPE cells, overexpression of Calpain-1 and Calpain-2 can inhibit ER stress and ER stress-induced apoptosis and autophagy.^298^ Taurine has been proven to protect against starvation-triggered ER stress by upregulating calpain.^298^

Gene therapy aimed at ER stress has developed rapidly. miRNA has been utilized in in vivo and in vitro experiments and is proven to be anti-vascularization, including MiR-106b.^209^ Stem cell and stem cell-derived exosomes are proven to act in subretinal fibrosis. The human umbilical cord MSC-derived exosomes secrete several miRNAs that are beneficial for the improvement of nAMD. For example, miR-27b inhibits subretinal fibrosis and increases cell migration, which cannot be achieved with VEGF therapy.^299^ Stem cell therapy has progressed in the treatment of dry AMD. Injection of human RPCs cocultured into the AMD region releases neurotrophic factors and differentiates into neurons and MG to supply the loss in dry AMD.^300^ RPE-derived exosomes, including α-Crystallin, can be transported into the eye and protect the RPE from apoptosis.^301^ However, whether this protein facilitates or inhibits VEGF in nAMD is controversial. Utilizing this exosome in the clinic requires further progress.

In conclusion, these drugs can be used for dry macular degeneration while enhancing the inhibition of angiogenesis by targeting the mechanism of ER stress in nAMD.

## ER stress and retinitis pigmentosa

**Fig. 6:**
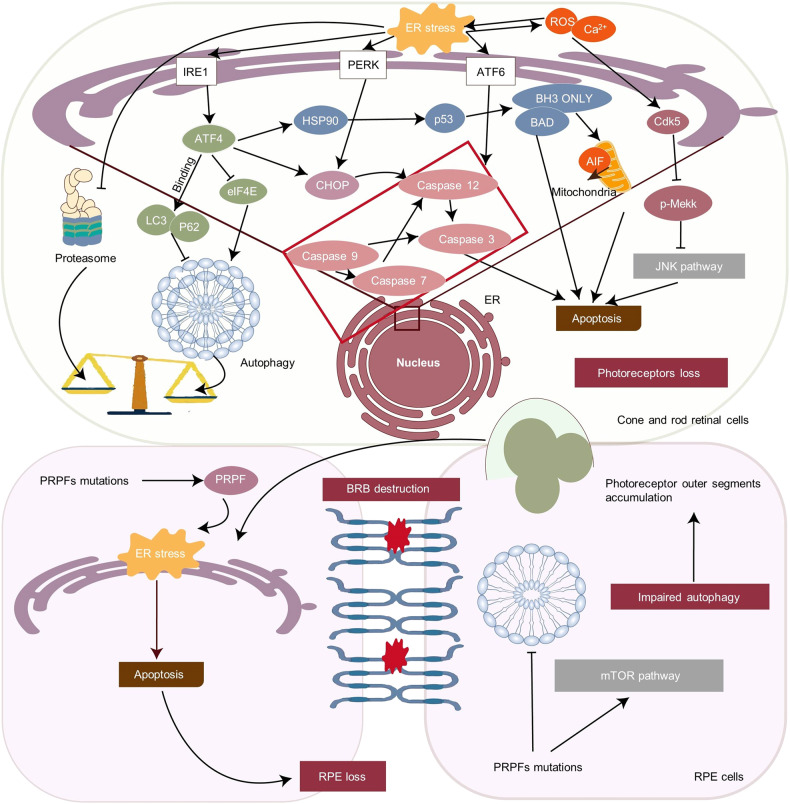
Involvement of ER stress in RP. The major pathogenetic process occurs in retinal cone and rod cells. The death of cone and rod occurs due to an imbalance between autophagy and ERAD. Impaired autophagy occurs through the P53-p38-MAPK-eIF4E cascade and is influenced by ATF4. In addition, ATF4 binds to key autophagy molecules LC3 and P62 to inhibit it. Proteasomes and lysosomes are responsible for clearing mutated protein, which is suppressed in RP. The ratio of autophagy: proteasome is decreased, which facilitates cone and rod cell death. ROS and Ca^2+^ can induce ER stress in RP. cdk5 is upregulated by ROS and Ca^2+^ and then stimulates the mekk1/JNK pathway, which promotes apoptosis. The IRE1α/ATF4/CHOP and ATF6 pathways influence caspase cascades and lead to apoptosis. ATF4 also influences p53 and then increases BH3 and Bad, which can lead to apoptosis directly and augment the mitochondrial permeability to facilitate apoptosis. RPE and blood-retinal-barrier (BRB) destruction are involved in the pathological process of RP. PRPF mutations cause the impaired autophagy and activated mTOR, which accelerate the photoreceptor outer segments. It is toxic for RPE. The accumulated PRPF protein accumulated in RPE induces apoptosis of RPE and destruction of BRB. The figure was created with BioRender.com (https://www.biorender.com/). RP retinitis pigmentosa, MAPK Mitogen-activated protein kinase, BRB blood-retinal-barrier, PRPF pre-mRNA processing factor, ERAD ER-associated degradation, ATF4 activating transcription factor 4, ATF6 activating transcription factor 6, eukaryotic translation initiation factor 2α (eIF2α), C/EBP-homologous protein (CHOP), PERK PKR-like ER kinase, IRE1 inositol requiring enzyme 1, XBP1 X-box binding protein 1

Retinitis pigmentosa (RP) is a hereditary disorder that is the most common form of inherited blindness and is characterized by the degeneration of rod and cone photoreceptors, with a prevalence of 1:3000 worldwide.^302,303^ The inherent features of RP can be autosomal dominant (30–40% of cases), autosomal recessive (50–60%), or X-linked (5–15%) traits. Although most RP cases are non-syndromic, 20–30% of RP patients develop non-ocular disease. The onset of RP can occur in childhood, teenage, adult, or older individuals. The classic pattern is that it is difficult for RP patients to adapt to dark and night blindness in adolescence and lose their mid-peripheral visual field in young adulthood. As the disease progresses, they will have weak peripheral vision, develop tunnel vision, and eventually lose central sight in their 60s.^302^ Cone and rod photoreceptors and RPEs are the most affected cells in RP. It has been shown that mutated and misfolded protein accumulation can induce ER stress in RPE, cone, and rod photoreceptors (Fig. 6).

### Involvement of ER stress in retinitis pigmentosa

#### Function of ER stress in photoreceptor cell death

Many gene mutations are involved in the development of RP (Table 1), and their pathogenesis is related to ER stress. However, each of these only relates to a small portion of RP. The most common mutation in RP is the rhodopsin gene (RHO) mutation, which directly results in photoreceptor degeneration and contributes to 25% of autosomal dominant retinitis pigmentosa (ADRP). Rhodopsin mutations, such as P23, R135w, Rh1^G69D^, T17M, and P53R, lead to the translocation of RHO protein from the cell membrane to the ER membrane and acceleration of ER stress.^304–307^ Among these, the P23 mutation is the most common.^308^ The harmful function of the mutant is reflected by protein accumulation and negative effects for WT RHO. The D1080N mutation in interphotoreceptor retinoid-binding protein, a key protein in photoreceptor survival, also prevents the transportation of proteins to the Golgi.^309^ A phosphodiesterase type 6 (PDE6) gene mutation is one of the main causes of ADRP. PDE6b encodes rod phosphodiesterase (PDE), which is an enzyme hydrolyzing cGMP to GMP in response to light and is only expressed in rod cells. Its mutation disturbs Ca^2+^ homeostasis and results in ER stress. Variants in interphotoreceptor matrix proteoglycans (IMPG2) have been reported in RP patients. IMPG is the matrix between photoreceptors and the RPE, which supports transduction, cell communication, and photoreceptor differentiation. Thus, IMPG2, resulting in matrix alteration, will cause retinal degeneration. In an IMPG2 KO mouse model, both rod and cone retinal cell degeneration happen at nearly the same time as ER stress activation and cell apoptosis.^307^

ER stress-induced OS plays a significant role in photoreceptor degeneration. Misfolded and unfolded proteins, including interphotoreceptor retinoid-binding protein and RHO, in photoreceptors can directly induce ER stress. ROS levels and Ca^2+^ levels increase dramatically, and then Mekk1 is phosphorylated, which induces cdk5 upregulation, and affects the JNK pathway and has a pro-apoptosis function.^305,310,311^ Rod photoreceptor death and retinal degeneration then occurs.^312,313^ In a retinal degeneration 10 model that possesses a PDE6 mutation and is widely used for RP studies, intracellular Ca^2+^ elevation-related ER stress occurs in the whole layer of the retina even before RP symptoms appear.^314^ As a result, ER chaperone S1R elevates in abundance to antagonize Ca^2+^.^314^ In the RGCs layer and cone photoreceptors, this early response can act as a protective response when rod cells are under attack. RNA-binding proteins pTDP-43 accumulates in the whole retina to form RNA stress granules. Over time, these harmful aggregates further activate ER stress, which leads to cell death other than rods, providing a possible mechanism for the death of cones, rod bipolar cells, horizontal cells, and RGCs followed by cone loss.

ER stress leads to mitochondrial dysfunction and photoreceptor mortality. p53 is responsible for the increase in the BH3-only protein BiK/Bad, which can augment the permeability of mitochondria, release apoptosis-inducing factor, and cause apoptosis.^315^ In RHO-mutated models, ATF4 coincides with p53 and is involved in photoreceptor apoptosis. Furthermore, mitochondrial dysfunction releases ROS and drives NLRP3 inflammasome activation in cone photoreceptors in an R23H model.^316^ In most cases, the loss of rod function is more severe than the loss of cone sensitivity. This shows cone retinal cell death is dependent on NLRP3 activation and that ER stress modulates the RIP1/RIP3/DRP1 axis of necrosis to induce degeneration.^316^

When mutated proteins, including RHO and interphotoreceptor retinoid-binding protein, accumulate, two clearance mechanisms, the ubiquitin-proteasome system or the autophagy-lysosome pathway, can be employed to deal with ER stress.^317^ ERAD is responsible for degrading accumulated protein. The IRE1α pathway is activated under ER stress and relies on functioning proteasomes and lysosomes to degrade the mutated and misfolded rhodopsin.^318^ Though ERAD is often beneficial for cell survival, it may be too effective and strong, resulting in cell death instead. However, in a *Rho*^*P23H/P23H*^ mouse model, the early stage of ERAD activation disturbs RHO homeostasis, which erases almost all RHO in photoreceptors, influences photoreceptor growth, and facilitates photoreceptor death. It can explain why some phenotypes of RP present early-onset photoreceptor degeneration and early vision damage. The ratio of autophagy and proteasome, or “A:P ratio,” influences the clearance of proteins, and the balance is broken when the A:P ratio is overwhelmed in the late stage of ER stress.^317^ P23H mice experience increased autophagy secondary to ER stress, which leads to proteasome insufficiency and increases retinal degeneration.^317^ The ATF4/CHOP pathway contributes to impaired autophagy in P23H-accumulated mice through the P53-p38-MAPK-eIF4E cascade and binding to LC3 and p62.^313^ The downregulation of p62 facilitates the upregulation of keap1 and excessive degradation of antioxidant nrf2. In IMPG2 KO mice, impaired autophagy, gliosis, and apoptotic cell death are induced by ER stress in both rod and cone retinal cells.^307^ Since activated autophagy is impaired and the autophagy flux is decreased, autophagy functioning in RHO accumulation is harmful to cell survival.^319^ However, in P10 models, ER stress arouses defective autophagy in the early stage of RP.^314^ In these cases, autophagy possesses a time-dependent dual function for retinal degeneration. Since ER stress plays a role in facilitating excess autophagy, PERK, independent of the ATF4 pathway, may exert a different role. When inhibiting PERK, RHO accumulation increases and rod retinal cell death is aggravated.^308^ In addition, in a Drosophila study, PERK inhibited massive autophagy of WT RHO by inhibiting the IRE1/XBP1 pathway,^320^ indicating that the PERK pathway facilitates photoreceptor survival in the early stage of ER stress.

#### Function of ER stress in retinal pigment epithelium cell death

Mutation of genes encoding pre-mRNA processing factors (PRPFs), including PRPF3, PRPF4, PRPF6, PRPF8, PRPF31, and SNRNP200, is the second most common cause of ADRP (Table 1).^321^ The retina is metabolically active tissue, and steady pre-mRNA splicing is required. Mutations in PRPFs may cause damage to retinal cell function by causing global spliceosome dysregulation and influencing important functional gene expression within the retina, including RHO, Abca4, and oxidative stress-related genes, resulting in RP. This explains why patients carrying PRPF mutations possess manifestations such as RPE atrophy and Brunch’s membrane thickening, like AMD pathological changes. In PRPF31^A216P/+^ mice, mutant PRPF31 protein mainly accumulates in the PRE’s cytoplasm, which can induce ER stress.^322^ Photoreceptor degeneration is secondary to PRE dysfunction and mortality.

Impaired autophagy, inadequate ERAD, and an activated mTOR pathway contribute to RPE and photoreceptor death in RP models. Ubiquitinations of misfolded proteins are transported to proteasomes for proteolysis. The HSP70 family, a protein chaperone family, is responsible for correcting aggregates folding. When the misfolded or deficient PRPF31 is overloaded, the HSP70 family protein binds to PRPF31 accumulated in the RPE and triggers ubiquitination.^323^ The photoreceptor outer segment cycle is also dependent on the RPE phagocytosis function, and the dysfunction of RPE will cause their accumulation in the RPE. To deal with inadequate ERAD, autophagy is activated, but the autophagy flux is lower than in normal cells, indicating impaired autophagy. Thus, another protein management system, the mTOR pathway, is activated. With time, the overloaded ubiquitination protein triggers ER stress in the RPE and results in caspase-3-involved apoptosis. This reveals one of the mechanisms behind Bruch’s membrane thickening in retinal PRPF mutations. In addition, the ubiquitination of the tight junctions between RPE accelerates BRB destruction. The NLRP3 inflammasome can be released from the RPE and function on photoreceptors, including cone and rod retinal cells. Therefore, photoreceptor outer segment accumulation, RPE atrophy, and BRB destruction lead to photoreceptors death in RP.

### Therapeutic targets for retinitis pigmentosa

There is no effective treatment for RP; only methods that slow its progress are effective; therefore, it is necessary to find effective drugs. There is evidence that oral vitamin A palmitate, vitamin E supplements, and docosahexaenoic acid can improve symptoms.^302^ Many mechanistically diverse approaches are under investigation, such as gene therapy, mutant genes (gene silencing by siRNA), stem cells, and small-molecule drugs (such as calcium-channel blockers), all of which have been used clinically but with limited influence.

Drugs targeting ER developed in RP are abundant. Many chemicals targeting ER stress can recover whole retinal cell function, including 4-PBA and TUDCA.^324^ 4-PBA used in RP can save the cone cell from necrosis and rod cell death, protect RGCs and prevent vision loss.^325,326^ In particular, 4-PBA recovers the mitochondrial genesis of rods to defend against cell death induced by mitochondrial dysfunction. Mutant rhodopsin-related cell loss can be suppressed by mTOR inhibition (rapamycin and PP242), AMPK activation (AICAR), and a peIF2α inhibitor (salubrinal).^308^ mTOR inhibition with rapamycin can restore the A:P ratio in RPE as well. Bilberry extract is antioxidant-rich, and oral administration can ameliorate light injury by suppressing photoreceptor apoptosis and attenuating ER stress in RGCs to reach a neuroprotective function.^312^ The HSP70 family, like HSPA4L, can colocalize with PRPF31 mutant aggresomes and translocate into nuclear function, which can be protective for RPE. Using a nanoparticle to deliver full-length genomic DNA of the rhodopsin gene can lead to a gain-of-function phenotype.^327^

Gene therapy offers profound insight into the future treatment of RP since most RP is associated with gene mutations. CRISPR/Cas9 is used in the research of RP mechanisms and treatments because of its high efficiency and accuracy. Since the RHO mutation is a gain-of-function mutation, thorough ablation of the mutation is more effective in ADRP. By selectively erasing the RHO mutation, mutant protein-induced ER stress will be ameliorated and symptoms will be relieved. AAV-CRISPR/Cas9 gene editing and ablation of pathogenic genes have been studied in the S334ter mutation. A study of editing genes using a neonatal rat model of P23H did not affect the survival of individuals and preserved long-term vision sight.^328^ If proven clinically safe, this technology can improve the quality of life of patients with ADRP from childhood. Silencing mutated RHO mRNA and facilitating WT RHO gene expression by double-stranded siRNA may be effective. For example, a bidirectional gene expression system consisting of a synthetic 75-mer-dsDNA to generate a hairpin loop siRNA and the other expressing the normal gene can be developed for silencing mutant rhodopsin by insertion.^329^

Stem cells can differentiate into specific cell types, and stem cell therapy can supply the normal cell loss in RP or release neurotrophic factors to support cell survival. RP patient-derived iPSCs with RHO mutations combined with genome editing can correct RP-related mutations.^330^ In one study, human iPSC-derived RPE cells were transplanted to a pig retina, which developed normal RPE morphology and facilitated photoreceptor survival.^331^ Transplantation of human embryonic stem cell-derived retinal organoids have been tested in RPE-loss rats and reveals the possibility of host organoid survival and integration of organoid and retina. The RGCs, photoreceptors, and RPE survival rates were improved, and organoids survived for more than 7 months.^332^ Furthermore, the combination of stem cells and gene editing technology can effectively treat RP, the kind of which begins with RPE cell death. Fibroblasts from RP13 patients who possess PFPR mutations are used as the somatic cell source for producing iPSCs. iPSCs whose P2301S mutation is corrected by CRISPR/Cas9 gene editing can be induced into RPE, which resembles WT RPE and possesses normal functions like phagocytosis.^333^ Though the injection of iPSC and directional homing ability have not been studied, they provide new ideas for RP treatment. Transplantation of MSCs or MSC-derived RPCs has been studied and proven to be effective in animal models. MSCs mainly express the RPE gene after transplantation into the retina. RPCs can express RGCs, RPE, and the photoreceptor genes and are distributed in different layers of the retina.^334^ Compared with iPSCs and MSCs transplantation, RPCs protect the whole retina tissue by differentiating and homing to RGCs and photoreceptors and releasing neurotrophic factors like BDNF, GDNF, IGF, CNTF, EG, FGF, and PDGF.^335^ However, when combining fetal RPE and MSCs, the whole retina can be saved.^336^

MSCs-derived exosomes can be a new option as cell-free therapy for RP therapy. Injecting MSCs, including bone marrow tissue and adipose tissue-derived exosomes, can suppress TNF-α and IL-1β, inhibit retinal cell apoptosis, and protect RGCs.^337,338^ RPE can release exosomes to maintain retinal homeostasis. Exosomes from RPEs release anti-inflammatory cytokines, suppress ER stress-induced inflammation and inhibit immune responses in vitro.^339^ Whether MSCs or RPEs-derived exosomes have a similar therapeutic effect to MSCs or iPSCs and the treatment of RP by inhibiting ER stress needs further study.

Optogenetic therapy has value in providing hope for vision restoration in advanced retinal degeneration.^340^ In RP, it usually refers to converting non-photosensitive retinal cells, usually bipolar cells or RGCs, into artificial photoreceptors, which is achieved by supplying these cells with a light-sensitive protein. Since the pathology of photoreceptor loss under ER stress is common, the advantage is that it can ignore the mutation type in RP and function in almost all types of RP by recovering light sensitivity. Opsin can be obtained from microbes and animals.^341^ It has been demonstrated that using a second-generation photoswitch for LiGluR, maleimide-azobenzene-glutamate 0, with peak efficiency at 460 nm, can restore rat vision.^342^

## ER stress and achromatopsia

Achromatopsia (ACHM) is a hereditary dystrophy that affects the central retina and is characterized by cone cell function loss, classically presenting with color blindness, photophobia, nystagmus, and decreased visual potential with a visual acuity of less than 20/200.^343^ Other symptoms include symmetric nystagmus with high frequency and low amplitude, which can occur in any direction. The actual signs of achromatopsia differ between patients owing to the varying degree of cone loss. It has been found that Ca^2+^ turbulence and UPR branch damage can result in ER stress, which leads to cone protein accumulation and cone death. We investigated the role of ER stress in the pathology of ACHM and ER stress-targeted therapy.

### Involvement of ER stress in achromatopsia

Gene mutations associated with cone cell dysfunction can cause ACHM (Table 1). Cyclic nucleotide-gated (CNG) channel mutations are the most common mutations in ACHM. CNG channel deficiency is characterized by elevated cellular cGMP levels and increased activity of cGMP-dependent protein kinase G, which leads to Ca^2+^ turbulence, cone degeneration, and impaired cone function, including CNG channel beta 3/ACHM3 and CNG channel alpha 3.^343,344^ Other gene mutations include PDE6H and PDE6C, which have been discovered in RP sections. Its mutation results in Ca^2+^ disturbance as well and is attributed to cone proteins, which are supposed to be located in the outer segment but misallocate in the inner segment, including cone proteins M-opsin, S-opsin, and cone phosphodiesterase subunit α' (PDE6C).^343^ An ATF6 gene mutation is directly related to automatic recessive ACHM, including a homozygous deletion covering exons 8–14, exons 2 and 3, and ATF6 c.970C>T (p.Arg324Cys), which are characterized by early vision loss.^345,346^ When ATF6 is stimulated, it can be activated but cannot activate its downstream pathways which are related to cell stress and proliferation. It is characterized by the absence of a foveal cone structure in these retinas.^347^ Currently, there is no effective treatment for achromatopsia. Studying the mechanisms of ACHM and finding new treatments is urgent and necessary.

ER stress is related to OS in cones and results in cone atrophy. Ca^2+^ turbulence is a contributor to ROS and OS, which can induce ER stress and act as a bridge in ER stress functioning in ACHM. The expression of 84 genes involved in unfolded protein binding, protein folding, ER-associated protein degradation, heat shock proteins, and enzymatic regulation of unfolded glycoproteins increases in a CNG channel deficient mouse model, which reveals the existence of ER stress in ACHM.^348^ Individuals lacking ATF6 experience severe ER stress-induced cell death and cone degeneration, which demonstrates the protective function of ER stress during the development of the retina in achromatopsia.^349^ In addition, ATF6 mutation not only influences the transcription activity of ATF6, or transportation of bZip, but also the IRE1 and PERK pathways, indicating an imbalance of ER stress in ACHM,^350^ leading to mislocalization of cone protein, Ca^2+^ turbulence, and ER stress-induced apoptosis.^351^ However, the specific mechanism underlying ER stress in ACHM requires further investigation.

Mitochondrial dysfunction is also a downstream effect of ER stress in ACHM. In retinal organoids generated from patients with ACHM who carried a homozygous ATF6 mutation, mitochondrial morphology changes and mitochondrial respiratory complex gene dysregulation can be identified.^352^ ER stress correlates with mitochondrial dysfunction, which has been proven in the retina. ATF6 mutation might cause an imbalance in UPR and result in ER stress.

### Therapeutic targets for achromatopsia

Owing to the intimate relationship between ER stress and ACHM, we can determine some target treatments aimed at relieving ER stress. In patients with ATF6 mutations, using drugs to increase the expression of this protein may save cone cells in the retina. It has been proven that ATF6 can be activated in two separate pathways: the protein toxicity activation pathway and the lipotoxic activation pathway, including specific sphingolipids, dihydrosphingosine, and dihydroceramide.^353^ It offers a unique way to treat ACHM with the ATF6 mutation through upregulating dihydroceramide by fenretinide, which facilitates ATF6 only in its normal function but not ER stress. Additionally, the selective ATF6 agonist AA157 can increase transcription in ACHM without effects on other UPR pathways.^349^ As for mutations in CNG dysfunction and PDE6 defects, recovering Ca^2+^ homeostasis and cGMP levels are effective ways to relieve ER stress. cGMP/protein kinase G regulates RyR2 expression. Downregulation of RyR2 can weaken cGMP-induced ER stress. For example, the deletion of IP3R1 and inhibition of ryanodine receptors, which control calcium efflux from the ER and are proven to facilitate ER retrotranslocation in Cnga3^−/−^/Nrl^−/−^ cone cells.^347^ Inhibition of protein kinase G by Rp-8-Br-cGMPS and KT5823 nearly completely abolishes channel upregulation in CNG channel deficiency.^348^

Gene therapy for ER stress in mutated cones has great potential. Clinical trials aimed at ACHM Cnga3 and Cngb3 have been successfully conducted, and short-term safety has been evaluated.^354^ AAV-delivered defect genes or neurotrophic factors can be considered. Subretinal injection of CNG cDNA packaged with AAV recovers cone function and vision.^355^ In late-stage CNGB3-achromatopsia, AAV-delivered CNGB3 cDNA and neurotrophic factors CNTF by subretinal injection to help restore cone function.^356^ CRISPR/Cas9 can be used in the ablation of mutated genes such as CNG, PDE6, and ATF6. However, the off-target effects have risks for patients. This can be avoided by using CRISPR/Cas9-mediated gene editing to correct single-nucleotide mutations for PDE6.^357^

## ER stress and cataracts

Cataracts refer to lens opacification and remain the leading cause of blindness in middle- and low-income countries,^358^ affecting approximately 94 million people worldwide. In 2020, about 15 million people over the age of 50 worldwide will be blind due to cataracts, and about 79 million will have moderate-to-severe visual impairment.^359^ Currently, there are various classification systems for cataracts. Here, cataracts are classified by the anatomical position of opacity as nuclear cataract, cortical cataract, posterior subcapsular cataract, and mixed cataract, with nuclear and cortical cataracts being the most common. Nuclear cataract lesions present as yellow-brown opacification of the fetus and adults on the lens, causing blurry vision, loss of color, sensitivity, and myopic shift, and the cornea presents as spoke cortical opacity with the same lens fiber shape, which causes astigmatism, monocular diplopia, and glare and halos around lights. Risk factors include smoking, genetic factors (such as KCNAB1 and CRYAA), alcohol consumption, and ultraviolet-B light exposure.^358^ Studies have shown that different metabolic pathways are involved in the formation of cataract lesions, such as elevation of the coproporphyrinogen pathway, dysregulation of crystal protein synthesis, lactose, etc., and cause pathological damage through yellow-brown opacity formation via ER stress of lens epithelial cells.

## Involvement of ER stress in cataracts

Genetic factors account for 35% of the variation in the progression of nuclear cataracts (Table 1). The most frequent transmission is autosomal dominant, whereas autosomal recessive or X-linked transmission can also occur.^358^ Some Wolfram syndrome patients possess dominant mutations in WSF1, including p.Glu809Lys, p.Glu830Ala, and p.His313Tyr. They can present as neonatal/infancy-onset diabetes, congenital sensorineural deafness, or congenital cataracts. These WSF1 mutations encode unfolded WSF1 proteins and accumulate, which induces ER stress (Table 1). EPHA2 encodes the Eph tyrosine kinase receptor family, which functions in epithelial homeostasis. Mutations and variants of EPHA2, including c.2819C>T, c.2915_2916delTG, rs7543472, and rs11260867, are related to age-related cataracts and congenital cataracts.^360,361^ In EPHA2 mutated mice, accumulation only results in moderate ER stress and UPR, which are protective, and the opacity is induced by glutathione (GSH) imbalance and fibroblast function turbulence.^362^ Calnexin is the lens fiber cell gap junction protein, in which connexin 46 and connexin 50 will result in cataracts.^363^ Mutations in connexin 46 and connexin 50 lead to junction and circulation lens damage, which results in Ca^2+^ elevation, precipitation, and biomineralization. Lens transparency decreases and can precipitate with proteins. Different connexin50 mutants induce ER stress, which plays different functions for cell survival or death that depend on downstream target genes.^364^

Epigenetic modifications, including DNA methylation, are regulated by ER stress in the development of cataracts. It is thought that DNA methylation can be inherited in somatic cells at the onset of embryonic development. The failure of DNA methylation can result from passive demethylation by Dnmt1, Dnmt3a, and Dnmt3b following DNA replication or from active enzymatic removal of 5 mC by ten-eleven translocation 1, activation-induced cytidine deaminase, and thymine-DNA glycosylase, independent of replication.^365^ Under ER stress stimulation, including valproic acid and sodium selenite, or risk factors Hcy for age-related cataracts, enzymes in passive demethylation and active demethylation of lens endothelial cells (LECs) are significantly upregulated.^366–369^ Methylglyoxal treatment induces ER stress in LECs, and the promoter DNA methylation status of keap1 is significantly lost, which causes dysregulation of the antioxidant system (nrf2), inflammation, and cell death in LECs.^366,368^

Cataractogenic stress, including alcohol, lipids, and hypoxia, generates misfolded protein conformations through the interaction of ER stress and OS. Insoluble crystalline is involved in the accumulation of LECs, which results in opacity.^362,370^ The lens is in a hypoxic environment, and factors such as diabetes will lead to increased anaerobic respiration of lens epithelial cells, which induces ER stress.^371^ At first, mild ER stress and ATF4 can act with nrf2, and the expression of ARE-dependent phase II antioxidant and detoxification enzymes increases, which reduces OS and ROS generation.^372^ Then, persistent ER stress induces superfluous ROS by upregulating keap1 and downregulating nrf2 resulting in LEC death and differentiation into fiber cells.^372^ The neolens fiber cells possess incomplete nrf2 systems and prefer to produce insoluble crystallin and misfolded proteins under ER stress, which can be seen as opacity. Furthermore, Ca^2+^ levels in the opaque part of cortical cataracts are higher than those in the transparent part, which can result in chronic UPR and is also a consequence of ER stress. Chronic UPR activates ERAD and m-calpain and then degrades sarco/ER Ca^2+^-ATPases and plasma membrane Ca^2+^-ATPases (PMCA).^365^ Thus, Ca^2+^ in the lens continues to rise and moves toward the lens fiber cells. Excessive ER stress and accompanied OS lead to massive Ca^2+^ elevation and activate caspase-12 and caspase-3-induced apoptosis.^373^ In Connexin50D47A mutant mice, gap junction damage between LECs will result in Ca^2+^ elevation.^364^ For mild ER stress and UPR for homozygous lenses, only the cell survival pathway PERK/ATF4/Trib3- IRS2 is activated, indicating the effect of ER stress is not static and related to the types and functions of different cataract-related mutations.

LECs go through epithelial–mesenchymal transition (EMT) induced by ER stress. Under ER stress, lens epithelial cells reduce the expression of epithelial genes and transform into fibroblasts in vitro.^374^ TGFβ facilitates the EMT process and has been proven to have a relationship with ER stress.^375^ After cataract surgery, remaining lens epithelial cells tend to proliferate excessively and result in fibrotic posterior capsular opacification (PCO). Postoperative stress-induced ER stress strengthens lens epithelial cell migration and EMT, which damages the lens conformation, explaining the development and pathology of PCO.

The interaction of autophagy and ER stress plays an important role in the pathogenesis of PCO. Under sulforaphane treatment in LECs, ER stress elevates ROS and activates the Erk pathway, both of which can contribute to autophagy.^376^ Combining ER stress and autophagy, lens epithelial cell organelles are impaired, and the recovery process cannot catch up, leading to apoptosis. Considering the role of ER stress in EMT, it can be concluded that the activation of mild ER stress is beneficial for PCO patients since it can inhibit the growth of lens epithelial cells.^375^ Therefore, ER stress plays a dual function in PCO, dependent on its degree.

### Therapeutic targets for cataracts

Surgery is usually performed to treat cataracts. However, the pharmacological prevention of cataracts is an area of future research. Regarding previously described treatments, sulforaphane can promote ER stress and relieve cataract symptoms.^376^ 4-PBA, a protein chaperone, can be used in calnexin mutant congenital cataracts since it can recover the misfolded calnexin and restore the junction between cells. Also, in PCO, 4-PBA has been found to prevent EMT of LECs and reduce fibroplasia and opacity.^377^ (+)-Pentazocine, a S1R agonist, has been administered to LECs in vitro.^378^ It can relieve ER stress, OS, and apoptosis, which can make it a potential drug to slow the progression of cataracts. Since oral or injected drugs do not target specific regions in the retina, iontophoresis is an innovative noninvasive strategy to deliver drugs through the anatomic barriers.^379^

## ER stress and uveal disease

### ER stress and uveitis

#### Involvement of ER stress in uveitis

Gene mutations play an important role in uveitis, in which ER stress is associated with pathology. Nucleotide-binding oligomerization domain containing 2 (NOD2) variants, including p.R334W, p.R334Q, p.E383K, p.G481D, p.W490S, p.M513T, p.R587C, and p.N670K, have been found in Chinese populations to be gain-of-function mutations and pathological in uveitis, especially for Blau syndrome. Blau syndrome is an early-onset autoinflammatory and autosomal dominant disorder typically characterized by a triad of uveitis, granulomatous polyarthritis, and skin lesions.^380^ The incidence is estimated to be 0.05 per 100,000 people per year.^381^ The NOD2 mutation has been found to directly induce aberrant autoactivation of NF-κB signaling and is attributed to a persistent induction of proinflammatory cytokines, including IFN-γ, IL-6, and IL-17.^382^ It has been demonstrated in many infection models as a direct ER stress inducer; NOD2 links ER stress with inflammation.^383,384^ Inflammation induced by IRE1α depends on the activation of NOD2, which activates NF-κB to induce inflammation. Similarly, in posterior uveitis, mutations of NOD-like receptor family genes (NOD2, NLRC4, NLRP3, and NLRP1) are detected to be gain-of-function in uveitis patients and are associated with ER stress and inflammation.^385^ For example, NLRP3 can be activated under ER stress and then recruit caspase-1, which initiates the pyroptosis pathway, and caspase-3, which induces apoptosis.^386^ R243L mutation in calpain-5 increases the catalytic activity of calpain.^387^ Under ER stress at the early stage, mitochondrial calpain-5 is activated first and cleaves caspase-4 to activate cell-programmed death and inflammation.

Behçet syndrome (BS) is an inflammatory disorder characterized by recurrent oral and genital ulcers, skin lesions, and uveitis. A comparison of blood samples from BS patients with those of healthy individuals revealed decreased ER stress-related proteins, such as HSPA8 and GRP78/BiP, but increased CHOP and ATF4, indicating dysregulation of the protein-folding mechanism and ER stress involved in BS pathogenesis.^388^ In acute anterior uveitis patients, ER stress increases proinflammatory cytokine levels.^389^

#### Therapeutic targets in uveitis

Since ER stress interacts with inflammation in uveitis, treatment targeting ER stress can alleviate the inflammation, which is the most significant pathology in uveitis. Using TUDCA or IRE1α inhibitors can relieve the inflammation induced by the IRE1/NOD2 pathway and NLRP3-induced pyroptosis and apoptosis.^383,385^ Galectin-3, a 31-kDa chimeric lectin characterized by a binding affinity for β-galactose-containing carbohydrates, can ameliorate the intestinal phenotype of BS via downregulation of ER stress and NLRC4 activation in BS.^390^ The concrete treatment effect on uveitis needs more investigation. Mesalazine is the most broadly utilized medication for mild to moderate ulcerative colitis and has been studied for adverse drug effects and control.^391^ Mesalazine can inhibit the transcription of proinflammatory and ER stress-associated cytokines and markers in BS.^389^

### ER stress and uveitis melanoma

Uveal melanoma (UM) is the most common intraocular malignancy that arises from melanocytes in the iris, ciliary body, or choroid.^392^ UM accounts for an estimated 5.5% of primary melanomas.^393^ Patients can be asymptomatic, and the most common clinical manifestations of primary UM include blurred vision, photopsia, floaters, and visual-field loss.^394^ Though current optimal treatment or surgery for most UM can be effective, up to 50% of patients possess metastatic symptoms with liver, lung, skin, and bone whose median overall survival is about 13.4 months.^395^

While patients of cutaneous melanoma harbor mutations in BRAF, RAS, and NF1 related to MAPK activation, few UM patients possess these mutations, but rather mutations in the G-protein α-subunit (Table 1).^396^ Uveal melanomas display MAP-kinase activation through the GNAQ^Q209L^ mutation. GNAQ operates downstream of several G protein-coupled receptors (GPCRs) that are important in melanocyte homeostasis and neoplasia and act as risk links between nevus of Ota and uveal melanoma.^397^ GNAQ and GNA11 encode an α-subunit of G-protein, which plays an important role in the conversion of GDP to GTP, and their mutation maintains the continuous activation of G-protein, which can activate Gα including activation of the MAPK, PI3K–Akt–mTOR, and Hippo pathways.^392^ The EIF1AX mutation has been significantly detected in UM, which affects the DNA methylation of cluster 1 tumors, in a multiplatform analysis of 80 UM identifiers.^398^

#### Involvement of ER stress in uveal melanoma

ER stress is involved in UM development. By single-cell sequencing of UM, ER stress and the UPR-related genes are shown to be expressed in UM tumor tissue. EIF1AX, which encodes EIF protein, is beneficial for tumors escaping immunological surveillance, and its mutation is attributed to the long latency and low metastatic rate of UM.^399^ Another study found that EIF1AX can predict the progression of UM and be used as the subtype standard.^398,400^ Analysis of 80 UM identifiers showed that ATF6 and XBP1 were activated upstream.^401^ Altogether, ER stress is part of UM’s progress.

Hypoxia broadly exists in the tumor microenvironment as a consequence of rapid cancer cell proliferation. In the UM microenvironment, hypoxia-related genes like BAIAP2L2, CTNNB1, EDNRB, HES6, LGALS1, PPM1K, PROS1, S100A4, and SYNPR can effectively predict the progression of UM.^402^ For example, HES6 enhances primary UM cell mobility and invasive characteristics.^402^ Hypoxia would increase the expression of the hypoxia-inducible factor 1 alpha subunit gene and its target gene, VEGF.^403^ As aforementioned, ER stress can induce the expression of VEGF and contribute to neovascularization. ER stress might be related to the development and prognosis of UM through the HIF1α/VEGF pathway.

Immune cell infiltration can be seen in the tumor microenvironment. It has been demonstrated that among 22 kinds of immune cells, the infiltrating proportions of CD8^+^T cells, follicular helper T cells, gamma and delta T cells, and activated natural killer (NK) cells were significantly higher in high-risk UM patients, while those of memory resting CD4^+^T cells, naive B cells, activated mast cells, resting mast cells, monocytes, and resting NK cells were significantly higher in low-risk UM patients.^402^ In CD8^+^T cells, ER stress and UPR may be cell-extrinsic regulators of immunity, and ER stress regulates DC cells by transmitting types of polarization and proinflammation, which promote the production of proliferation-deficient T cells.^404^ This phenomenon may explain the high correlation between CD8^+^ T cell infiltration and high-risk UM.

ER stress might be potentially related to UM metastasis. MMPs are overexpressed in UM tissues compared to normal tissues, including MMP1, MMP9, MMP10, MMP11, MMP13, MMP14, and MMP17. MMPs can degrade various protein components in the ECM, destroy the histological barrier of tumor cells, and affect tumor migration, invasion, metastasis, and angiogenesis. ER stress can be cytoprotective and drug-resistant in UM.^405^ MMPs expression can serve as a biomarker of UM prognosis. Meanwhile, in mouse mammary gland tumor models, MMP11 promotes tumor growth through ER stress activation and metabolic rewiring.^406^ Overexpression of the pro-survival BCL-2 family members has been broadly discovered in cancer and also in primary UM. The effect of BCL-2 inhibitors in UM can be weakened by ER stress.^407^

#### Therapeutic targets for UM

Two types of management for localized UM are globe-preserving therapy and enucleation. Globe-preserving therapies can broadly be divided into radiation, surgical, and laser therapy.^392^ Considering the involvement of neovascularization in tumor metastasis and growth, tumor-targeted approaches, including immunotherapies, are under research. Targeting factor VII, ICON-1 is a structural variant of human factor VII being developed by Iconic Therapeutics and in Phase I study. ICON-1 contains virus-like particles that selectively bind to cancer cells and infrared-activated molecules that destroy tumor membranes when activated by an ophthalmic laser.^392^ However, current effective therapies for metastasis are limited, and the prognosis is poor. Some studies focus on the MAPK pathway and epigenetic modification with an HDAC inhibitor. Regarding the complicated pathology and poor prognosis of late-stage UM, new, effective approaches are needed.

ER stress has been proven to be a novel target in UM treatment. Direct intervention on ER stress might slow tumor growth and improve prognosis. Though not previously studied in UM, 4-PBA, a selective ER stress inhibitor, has been proven to be effective for BRAF-mutated melanoma.^408^

ER stress possesses a dual function in UM cell survival. Some research has proved that ER stress plays an anticancer role in UM and contributes to choroidal melanoma cell apoptosis. Current evidence shows that choroidal melanoma is insensitive to many commonly used chemotherapeutic drugs, and properly utilizing ER stress can enhance the chemotherapy effect.^409^ Cisplatin is a commonly used chemotherapy drug that binds to DNA and results in cell death. The synergistic effect of cisplatin plus pemetrexed contributes to the induction of simultaneous extrinsic and intrinsic apoptosis.^410^ Intrinsic apoptosis induction is controlled by ER stress through the CHOP/NOXA/Mcl-1 pathway.^410^ Lithium chloride promotes apoptosis through the CHOP/NOXA/Mcl-1 pathway, which increases NOXA and decreases the anti-apoptosis molecule Mcl-1.^411^ Using BH3-mimics to inhibit BCL-2 can be considered a treatment strategy for UM. Exerting ABT-263 (Navitoclax) in the treatment of UM is antiproliferative and blocks the PERK pathway while promoting apoptosis, proving the cytoprotective effect of ER stress.^407^

Immunotherapy can also be enhanced by combining it with ER stress enhancement or reduction. Immunogenic cell death can be evoked by ER stress-induced ROS. Photodynamic therapy and photothermal therapy are commonly used immunotherapies for tumors that rely on ROS in cells. Based on ER stress function, combining targeted ER stress therapy with photodynamic therapy and photothermal therapy enables sustained ROS levels and continuously induces immunogenic cell death.^412^ An ER-targeting nanosystem consisting of ER-targeting pardaxin peptides modified with indocyanine green conjugated hollow gold nanospheres together with an oxygen-delivering hemoglobin liposome has giant prospects.^412^ Reversing activated ER stress in CD8^+^ T cells helps the production of antitumor CD8^+^ T cells. For example, in human colon cancer, inhibition of stearoyl-CoA desaturase 1 improves antitumor properties by regulating β-catenin signaling in cancer cells and ER stress in T cells.^413^

## ER stress and ocular surface diseases

### ER stress and corneal dystrophy

Corneal dystrophies have typically been referred to as a group of inherited disorders that are usually bilateral, symmetric, slowly progressive, and unrelated to environmental or systemic factors. In the ICD3 classification of corneal dystrophy, a modified anatomic classification is proposed consisting of (1) epithelial and subepithelial dystrophies, (2) epithelial–stromal TGFBI dystrophies, (3) stromal dystrophies, and (4) endothelial dystrophies.^414^ Most corneal dystrophies are characterized by progressive opacification of the cornea and eventually a reduction in visual acuity.

#### Involvement of ER stress in corneal dystrophy

ER stress has been proven to be involved in various types of corneal dystrophies, including macular corneal dystrophy (MCD), granular corneal dystrophy type 2, congenital stromal corneal dystrophy, Schnyder corneal dystrophy, congenital hereditary endothelial dystrophy, and Fuchs endothelial corneal dystrophy. Corneal dystrophies possess different mutations that code for unfolded proteins, produce accumulations of mutant glycoproteins or proteins, overexpress metabolisms, and result in ER stress (Table 1). Enlarged rough ER can be seen in corneal endothelial cells, which reveals impaired ER function.^415,416^ In addition, the overload of ECM proteins in the stroma induces aggresomes and ER stress in the corneal endothelium.^417^ Next, we will discuss the role of ER stress in different coraeal dystrophies.

MCD is a rare autosomal recessive disorder, usually evident in childhood or adolescence, and is clinically characterized by the formation of a diffuse and fine symmetric clouding in the central corneal stroma that extends to the periphery and eventually involves the entire thickness of the cornea, leading to severe bilateral visual disturbance.^414,418^ Deposition of abnormal glycosaminoglycan material in the stroma can be observed in an irregular position, as an infiltration of the adjacent corneal structures, including Bowman’s layer, Descemet’s membrane, and the endothelium.^419^ The pathology of MCD has been shown to entail a deficiency in acid mucopolysaccharide metabolism localized in the keratocytes (corneal fibroblasts), whose ER is surrounded by glycosaminoglycan accumulations.^415,419^ The carbohydrate sulfotransferase gene, encoding an enzyme designated corneal *N*-acetylglucosamine-6-sulfotransferase (C-GlcNAc6ST), is identified in MCD pathology.^420^ ER stress markers like GRP78 and CHOP are expressed when ER stress is active.^415,421^ In cell models transfected with c.463-464del and c.250_272del carbohydrate sulfotransferase gene mutants, ER stress-induced apoptosis was activated.^421^

Furthermore, the interaction between ECM and ER stress can play a role in the pathogenesis of corneal diseases as well. Congenital stromal corneal dystrophy is a human genetic disease characterized by corneal opacities beginning shortly after birth and is associated with a decorin gene mutation.^422^ Decorin is a multifunctional small leucine-rich proteoglycan that interacts with collagen fibrils and regulates fibrillogenesis in ECM assembly. Its mutation will cause a deficiency in the correct organization of the corneal stroma. Mutant decorin has an intrinsic deficiency in entering the Golgi apparatus, which causes the accumulation and nonsecretion of decorin in keratocytes.^423^ The intracellularly ER-retained decorin protein core lacking the C-terminal ear repeat causes ER stress.^423^ Granular corneal dystrophy type 2 is an autosomal dominant disorder caused by an arginine to histidine substitution at codon 124 (R124H) in the transforming growth factor β-induced protein (TGFBI) gene.^424^ Mutant-TGFBI protein is an age-related progressive deposition in the corneal epithelia and stroma, and its secretion via the ER/Golgi-dependent secretory pathway is delayed.^425,426^ TGFBI protein accumulation in fibroblasts results in protein homeostasis disturbance and ER stress.^427^ Fuchs endothelial corneal dystrophy is characterized by progressive alterations in endothelial cell morphology, excrescences, thickening of the endothelial basement membrane, and cell death, which causes corneal edema and vision loss. The Col8a2^Q455K/Q455K^ mutation results in alpha-2 collagen VIII structural modification, activated UPR (including the IRE and PERK pathways), and intrinsic apoptosis in mouse endothelium.^428^ Increased TGFβ was discovered in Fuchs endothelial corneal dystrophy which directly increased MMP activity and promoted ECM accumulation and apoptosis.^417^ The accumulation and overexpression of proteoglycans and ECM, including fibrin and collagen I, help the formation of aggresomes in the endothelium, which act as induction of ER stress followed by cell death.^429^

Metabolite accumulation or metabolism pathway disturbance in corneal dystrophies is common, which may inhibit normal ERAD systems and affect ER stress. A Slc4a11 protein mutation has been detected in congenital hereditary endothelial dystrophy, where elevation of glutamine is a contributor to mitochondrial ROS and ER stress.^416^ Schnyder corneal dystrophy is associated with the UbiA prenyltransferase domain-containing protein-1 mutation, which regulates the ER-resident enzyme HMG-CoA reductase in normal condition.^430^ UbiA prenyltransferase domain-containing protein-1 is responsible for HMGCR ERAD, and its mutation will result in the persistent activation of HMG-CoA reductase and sterol production.^430^ Thus, the balance between ERAD and autophagy shifts to autophagy.

#### Therapeutic targets for corneal dystrophy

The current effective treatment for corneal dystrophy is corneal transplantation. Considering the risk of graft rejection and difficulty acquiring corneal donations, finding a new treatment is urgent. As for the important role of ER stress in the onset and development of corneal dystrophy, targeting ER stress can be considered a new method. Inhibiting ER stress with drugs can prevent corneal cell death. Reinforcing ERAD can effectively solve mutant protein accumulation. 4-PBA has been demonstrated to activate ERAD and accelerate TGFBIp degradation.^427^ Melatonin alleviates the IRE1α/XBP1 pathway and facilitates the activation of ERAD, which can save granular corneal dystrophy type 2 corneal fibroblasts.^431^ Reduction of ER stress and simultaneous facilitation of protein accumulation by autophagy is beneficial for survival; for example, the use of lithium in Fuchs endothelial corneal dystrophy.^432^

Appropriately reducing the inducers of ER stress, like the alleviation of OS and facilitating protein folding, can save cells from ER stress-induced death. Glutamine catabolism elevates ROS in Slc4a11^−/−^ cells and leads to mitochondrial dysfunction. Mitochondrial ROS quencher MitoQ can reduce BiP and GADD153 expression in Slc4a11 KO cells and mice.^416^ It has been successfully demonstrated that the coexpression of R125H and SLC4A11 facilitates mutant protein transportation to the cell membrane without affecting water flux.^433^ Antioxidative therapy like *N*-acetylcysteine can relieve ER stress.^434^ High-throughput assays allow scientists to screen drugs like glafenine and perhaps other non-steroidal anti-inflammatory drugs for helping SLC4A11 properly fold,^435^ and could also find more potential therapeutics for corneal diseases.

Extracellular vesicles have been broadly investigated in ocular diseases. MSC-derived extracellular vesicles have been verified for their treatment effect in an in vitro model of corneal dystrophy.^436^ MSC-derived extracellular vesicles contain targeted ER stress miRNAs for ER stress-related genes, including phosphorylated EIF2α, ATF4, and CHOP.^436^

### ER stress and keratoconus

Keratoconus (KCN) is a bilateral and asymmetric disease characterized by restricted cone-like bulging of the cornea, which leads to progressive thinning and steeping of the cornea, leading to irregular astigmatism and decreased visual acuity.^437^ The prevalence and incidence of KCN vary globally and are estimated to be between 0.2 and 4790 per 100,000 people and 1.5 and 25 per 100,000 people/year, respectively.^438,439^ Changes in degradation systems and OS can be related to ER stress in KCN pathology.

Genetic factors are essential in the development of KCN, and some of them are found to be related to ER stress in the cornea. Genetic analysis has revealed that ER stress markers and multiple translation initiation and elongation factors (EEF1A1, EEF1B2, EEF1D, EIF3F, EIF3H, and others) downstream of ATF4 are increased in the corneal proteomes of KCN, indicating the involvement of ER stress.^440^ Mutations in superoxide dismutase 1, including c.169+50 delTAAACAG, have been discovered in KCN patients.^441^ The protein encoded by SOD 1 can bind to copper and zinc ions, which is an enzyme for radical clearance whose mutation causes OS.^442^ As aforementioned, OS and ER stress each other, which may cause damage to the cornea. OS will cause peroxynitrite overexpression, causing prolonged oxidative injury. Peroxynitrite can induce ER stress through the depletion of ER-Ca^2+^ in endothelial cells.^443^ Increased ROS in cells might cause increased MMP, which can be the mechanism of corneal thinning.^444^ For example, in fibroblasts, the overexpression of SOD 2 increases the expression of MMP-1, -2, -3, -7, -10, -9, and -11.^445^ Also, ER stress has been found to elevate MMP expression in neurons, which offers the possibility of an interaction between OS and ER stress to facilitate MMP activation and corneal stromal degradation. TGFB1 is now proven to be involved in KCN, including the G535X.

### ER stress and keratitis

Incomplete eyelid closure and blinking cause corneal desiccation and epithelial barrier compromise, which leads to chemosis, erosion, melting, infectious keratitis, and even perforation.^445^ In the pathogenesis of exposure keratitis, air exposure induces ER stress in corneal epithelial cells and activates autophagy via the PI3K/AKT/mTOR signaling pathway.^446^ In a keratoconjunctivitis sicca model, ER stress markers, such as GRP78 and sXBP1, are increased, which destroys the ocular surface barrier and facilitates goblet cell death.^447^ The loss of goblet cell death will result in mucosal decrease and the destruction of the tear film, which is an important eye barrier. Herpes simplex keratitis is an infectious disease caused by herpes simplex virus infection of the cornea. Most herpes simplex viruses (HSVs) infecting the eyes are HSV-1, and some are HSV-2. ER stress is related to immune homeostasis. As aforementioned, STING is crucial for initiating innate immune responses and producing interferon regulatory factor 3 to defend against virus invasion.^212^ HSV-1 infection results in STING carbonylation and causes inhibition or deactivation of protein function.^212^ In *Mycobacterium bovis*, activation of STING/TBK1/interferon regulatory factor 3 is dependent upon ER stress.^448^ In *Brucella abortus*, UPR is necessary for STING activation and the production of IFN.^182^ Therefore, ER stress can be significant in defending against bacteria and viruses through STING/IFN1 activation.

Nowadays, antibiotic abuse results in bacterial resistance in many patients. In an attempt to address antibacterial drug resistance while avoiding toxicity toward mammalian cells, large lipids fraught with small nanoparticles encapsulating antibiotics have been developed and tested against gram-negative bacteria, in which lipids can integrate with bacterial membranes, and nanoparticles are released to kill bacteria.^449^

### ER stress and dry eye syndrome

Oxidative stress correlates with dry eye syndrome (DES) and the core mechanism can be tear-high osmotic pressure during inflammation.^450,451^ Under high osmotic pressure conditions, increasing ROS levels lead to ER stress in corneal epithelial cells and induce apoptosis.^450^ Goblet cells, which are integral components of the tear film, are the most important source of mucins and other proteins. In an interferon-γ-induced dry eye model, expression of GRP78 and XBP1s increased, especially in goblet cells, and contributed to cell apoptosis and inflammation.^447^

In Sjögren’s syndrome, ER stress activates and induces MMP9 activation through the FOX/MAPK pathway, which increases proteolytic activity and results in DES.^452^ Additionally, MAM functions in this syndrome, during which it can activate ER stress and inflammation.^453^ In aquaporin 5-deleted mice, the secretion of the lacrimal gland is injured, and there are symptoms of dry eye.^454^ Moreover, GRP78, CHOP, Caspase12, and BAX levels increase, indicating the occurrence of ER stress.^454^ High osmotic pressure induces dry eye through GADD34.^455^

## ER stress and myopia

Myopia refers to a condition often developed during childhood and early adulthood where excessive elongation of the eye results in images of distant objects coming into focus in front of the retina, leading to blurred distance vision.^456^ The global prevalence of myopia in 2010 was about 2 billion individuals, of whom 277 million had high myopia.^457^ The prevalence is estimated to increase to 4.76 billion individuals for myopia and nearly 1 billion for high myopia by 2050. The most common mechanism is excessive and progressive axial eye growth.^458^ Besides the elongated axial eye, thinning of the retina and sclera are also important pathophysiological changes in myopia.

### Involvement of ER stress in myopia

ER stress is directly involved in axial elongation. The ATF6 and PERK pathways, especially ATF6, can induce axial elongation, albeit with a tendency.^459^ ER stress plays an important role in scleral remodeling during the progression of myopia. The sclera’s ECM is mainly secreted by fibroblasts. Type I collagen is the major component of scleral ECM, and a decrease will weaken the scleral structural framework. It has been revealed that the decreased synthesis and increased degradation of sclera ECM contribute to sclera remodeling. The HIF-1α signaling pathway is highly activated in myopia, indicating a hypoxic environment.^460^ One of the mechanisms can be a decrease in choroidal blood perfusion, resulting in hypoxic conditions.^461^ ER stress signaling eIF2α is activated under HIF-1α inducement, which facilitates myofibroblast transdifferentiation and decreases collagen production (including α1 [COL1A1]).^460^ Furthermore, under ER stress, Col4a3, Col8a2, Col11a2, and Col15a1 are significantly upregulated, indicating ECM remodeling is regulated by ER stress.^458^ In an in vitro study, enlarged ER has been observed in fibroblasts, and ER stress is induced under deprivation to induce myopia, which facilitates COL1A1 and TGFβ at the early stage and decreases ECM synthesis at the late stage.^462^ Therefore, ER stress can be an adaptive response in myopia and compensate for ECM degradation via calreticulin-mediated collagen synthesis.^461^

### Therapeutic targets for myopia

Since hypoxia and ER stress correlate with each other in the development of myopia, they can be novel targets for myopia treatment. Salidroside and formononetin are the active extracts of traditional Chinese medicines that have been proven to resist hypoxia-induced injury separately in heart and retina neovascularization.^463,464^ By periocular injection of these drugs, the progression of myopia is slowed down, HIF-1α is decreased, and phosphorylation of eIF2α is declined as well, leading to COL1α1 restoration.^460^ Direct inhibition of ER stress by 4-PBA and TUDCA normalizes the vitreous chamber depth, and retinal thickness shortens in mouse eyes, which tends to shorten myopia progress.^459^ Gene therapy can be considered since simultaneous inhibition of PERK and ATF6 by CRISPR/Cas9 can slow axial elongation.

## Conclusions and prospects

ER is a quality-control organelle for proteostasis. Proteostasis disturbances will result in ER stress, which initiates an adaptive response called the UPR. In recent years, ER stress as a potential modulator in human diseases has been discussed broadly, which can affect ERAD, OS, mitochondrial dysfunction, autophagy, and metabolism. However, the concrete mechanisms of ER stress in different diseases have not been revealed. In most circumstances, ER stress is considered a pro-degenerative process for disease development. However, the different stages of UPR induced by ER stress have opposite functions, indicating the complicated role of ER stress. In this review, we discuss and conclude the molecular mechanisms and treatment targets of ER stress, focusing on ophthalmology, including POAG, AMD, RP, DR, UM, and other ocular diseases.

The notion that ER stress contributes to ocular diseases is commonly researched. The discoveries of mutations in genes that encode the UPR molecule, ER chaperones, misfolded proteins, and metabolisms in all types of ocular diseases indicate the importance of maintaining proteostasis for normal eye function. We point out that ER stress not only affects ocular disease homeostasis through misfolded protein accumulation but also correlates with epigenetic modifications, mitochondrial dysfunction, OS, and impaired autophagy. In concrete terms, ER stress modulates retinal degeneration and neovascularization, leading to pathological changes in glaucoma, AMD, DR, ACHM, and RP. Studies also suggest the involvement of ER stress in lens opacity, epithelial degradation, and ECM remodeling, which have implications for POAG, ocular surface diseases, and cataracts. Additionally, the role of ER stress in regulating the microenvironment underlines the potential effect of ER stress on tumorigenesis and inflammatory diseases like uveitis and keratitis. ER stress is involved in many pathways and plays a key role in many pathogenic processes. Since ER stress plays a complicated role in ocular diseases, further studies are required to elucidate this role.

ER stress is a promising and novel target for the treatment of ocular diseases (Table 2). Recently, several methods, including drugs, nanoparticles, gene therapy, stem cell therapy, and cell-free therapy, have been developed. Drugs, including chemicals, natural extracts, and novel nanoparticles, have been broadly investigated. For example, 4-PBA is an effective ER stress inhibitor that has been utilized in various ocular diseases like glaucoma and DR. Gene therapy has been widely studied, and technologies including CRISPR-Cas9 and ncRNA are innovative and effective. Using gene editing in RP is a hot topic, and some research has been in the clinical trial phase. Stem cell therapy and cell-free therapy provide new insights for treatments targeting ER stress in ocular diseases. As we discussed, TMSCs can differentiate into TM cells and are home to TM tissue, which can survive for months. Treatment targets for ER stress require further research before they can be used clinically. Whether ER stress is involved in ocular diseases and whole-body health remains unclear. Therefore, simply observing aspects of ER stress in ophthalmology is insufficient. Greater efforts are needed for systematic research on the molecular mechanisms of ER stress and the safety of interventions for UPR in ocular diseases. We hope that our review inspires future research into treatment targets for ER stress.

**Table 2:** ER stress is effective therapeutic target for ocular diseases

Disease	Agent	Facilitate ER stress (+)/inhibit ER stress (−)	Target	Pathway	Function in ocular diseases	Used in other fields	Reference
Glaucoma	4-PBA	−	Myocilin, MMP2, and MMP9	The whole ER stress pathway	Facilitation of myocilin outflow and degradation in TM	Heart, lung, and kidney injury	^152,153^
Glaucoma	4-Br-BnIm	−	GRP94	ER stress chaperone	Inhibition of GRP94 lower the levels of mutant myocilin and save RGCs	Null	^149^
Glaucoma	Astragaloside-IV	−	MMP3 and MMP9	The whole ER stress pathway	Rescue TGF-β2 induced ocular hypertension by relieving RM damage	In renal and cardiac diseases	^153^
Glaucoma	shp2	+	PERK	PERK pathway BDNK/TrkB pathway	Knock down of Shp2 relieve ER stress	Cancer for immunotherapy, human development disorders, and aggravating psoriasis	^122,472–474^
Glaucoma	CRISPR-Cas9	−	Myocilin/HRH1/CHOP/ATF4	The whole ER stress pathway	Remove MYOC mutation/alleviation of Ca^2+^ release/knockout UPR molecules to protect TM cells and RGCs	Null	^77,108,175^
Glaucoma	TMAO	−	Unfolded myocilin	ER chaperone	Facilitate myocilin outflow	In urethral cycle disorders for clinical use	^151^
Glaucoma	LDN-0060609	−	PERK	PERK-ATF4-CHOP	Relieve ER stress and protect TM cells	In Alzheimer’s disease	^150^
Glaucoma	siRNA	−	Mutant MYOC	The whole ER stress pathway	Repopulation of TM cells, prevention of RGCs loss, and recovery of IOP	–	^75^
Glaucoma	siRNA	−	PKR	The PERK/ eIF2α/ATF4/CHOP pathway	Relieve ER stress and protect RGCs	–	^176^
Glaucoma	Imidazolo-oxindole derivative	−	PKR	The PERK/ eIF2α/ATF4/CHOP pathway	Relieve ER stress and protect RGCs	–	^176^
Glaucoma	Human trabecular meshwork stem cells	−	TM tissue	The whole ER stress pathway	Relieve of ER stress, replication of endogenous TM cells, and the restoration of the ECM structure	–	^161,162^
Glaucoma	Induced pluripotent stem cells	−	TM tissue	The whole ER stress pathway	Relieve of ER stress and supplement of TM cells	Not used in clinic	^163^
Glaucoma	Mesenchymal stem cells	−	TM tissue	The whole ER stress pathway	Relieve of ER stress and supplement of TM cells and protection of RGCs	Not used in clinic	^164^
Glaucoma	Exosomes derived from bone marrow mesenchymal stem cells	−	TM tissue	The whole ER stress pathway	Protection of TM cells from ER stress	Not used in clinic	
Glaucoma	Non‐pigmented ciliary epithelium derived exosomes	−	ECM degradation and	WNT/β‐catenin pathway and nrf2 downstream and the whole ER stress pathway	Decreased ECM and ER stress in TM tissue, and alleviation of inflammation	–	^167^
Glaucoma	Adipose-derived stem cells	−	TM tissue	The whole ER stress pathway	Protection of TM cells from ER stress	–	^166^
Glaucoma	Combining stem cell therapy with superparamagnetic iron oxide nanoparticles	−	TM tissue	Relieve the whole ER stress pathway	Drive the homing of stem cell and preserve TM tissue	–	^165^
Glaucoma	Amoxapine/desloratadine/maprotiline	−	RXR	PERK-ATF4-GADD34	Relieve ER stress	In relapsing–remitting multiple sclerosis, hematoma, diabetes, and lung cancer and	^108^
Glaucoma	Valdecoxib	−	COX2	PERK/ATF4/CHOP pathway	Reduce RGC apoptosis	In the treatment of osteoarthritis (OA) and arthritis	^177^
Glaucoma	KUSs	−	ATPase	Autophagy	Protection of RGCs, amacrine cells, and photoreceptors	Ischemic neuropathy	^170^
Glaucoma	p58^IPK^ overexpressed by AAV	−	Neurotrophin	ER protein chaperon	Elevation of RGCs survival rate through refolding protein and inhibition of ER stress	–	^115^
Glaucoma	Resveratrol	−	RXRs and HDAC1	The whole ER stress pathway	Reduce ER stress-induced RGC apoptosis	–	^178^
Glaucoma	Tubacin	−	HDAC6	The whole ER stress pathway	Reduce oxidative stress and ER stress to protect RGCs	In the treatment of bipolar disorder and epilepsy	^134^
Glaucoma	Valproate	−	HDAC	Bip and CHOP	Reduce oxidative stress and ER stress to protect RGCs	In the treatment of bipolar disorder and epilepsy	^136^
Glaucoma	Rapamycin	−	Autophagy	mTOR pathway	Restore autophagy caused by OPTN mutation and relieve ER stress	–	^102^
Glaucoma	Trehalose	−	Autophagy	Autophagy independent of mTOR pathway	Restore autophagy caused by OPTN mutation and relieve ER stress	–	^102^
Glaucoma	Nanoparticles	−	ER stress and oxidative stress	ROS and the whole ER stress pathway	Inhibit ER stress and oxidative stress at the same time	–	^179^
Glaucoma	Pentazocine	+	S1R	IRE1/XBP1 pathway	Upregulate the protective IRE1/XBP1 pathway and reduce the other two to protect RGCs	–	^113^
Glaucoma	BDNF/MANF/rhNGF	−	Neurotrophin	The whole ER stress pathway	Relieve ER stress to protect RGCs	–	^117,120,181^
Diabetic retinopathy (DR)	4-PBA	−	The whole ER stress pathway	The whole ER stress pathway	Inhibit ER stress and protect retinal neuron	Heart, lung, and kidney injury	^152,153,187^
DR	TUDCA	−	TGR5	The whole ER stress pathway	Alleviate ER stress by inhibiting Grp78 translocation and reduce VEGF that protect inner BRB	–	^249^
DR	Nobiletin	−	GADPH translocation	The whole ER stress pathway	Protect Muller glia and recover VEGF/PEDF, which protect iBRB	–	^207^
DR	Ghrelin	−	GHSR-1a	PERK pathway	Inhibit ER stress in endothelial cells to protect inner BRB	–	^250^
DR	Astragalus polysaccharide	−	miR-204	miR-204/SIRT1 axis	Alleviate inflammation and reduce the apoptosis of RPE cells	In tumor treatment	^189,244,245^
DR	Lactucaxanthin	−	ER stress, oxidative stress and inflammation	The whole ER stress pathway	Decrease vascular leakage due to rescue the destructed tight junction between RPE cells	For antioxidants, antidiabetic substance, and anticancer drugs	^208,233,246–248^
DR	CRISPR-Cas9 technology	−	IRE1α	IRE1α/XBP1STING pathway	Protection of endothelial cells to protect iBRB	–	^213^
DR	siRNA	−	TCF7L2	ATF6 pathway	Protect vascular endothelial cells and iBRB	–	^217^
DR	P58^IPK^	−	neurotrophin	PERK/eIF2α pathway	Protect vascular endothelial cells by inhibiting VEGF production	–	^251^
DR	Liraglutide	−	Trx-ASK1	Trx-ASK1complex/ER stress	Protect RGCs by inhibiting ER stress	In T1DM, T2DM, and obesity	^475,476^
DR	Sulforaphane	−	AMPK	Inhibit ER stress by AMPK pathway	Protect retinal cells including photoreceptors by reducing inflammation, oxidative stress, and ER stress	In age-related diseases and neuroinflammation diseases	^254^
DR	Melatonin	−	PERK pathway and Bip	PERK pathway	Prevent RGCs death via inhibiting ER stress	In the control of the circadian rhythm, immune enhancement, and antioxidant, anti-aging, and antitumor effects	^256^
DR	Stem cell therapy	−	Adipose-derived mesenchymal stem cells	Combining the neuroprotective effect with melatonin	Neuro protection	–	^256^
DR	Chrysin	−	UPR and AGE-RAGE	Reduction of UPR and activation of AGE-RAGE	Save the BRB by rescuing RPE and reduction of VEGF	In degenerative disorders and provides cytotoxic and anti-inflammatory functions	^194^
DR	Penicillamine	−	Elevated copper	The whole ER stress pathway	Protect BRB by saving RPE via alleviating ER stress and MFN2	–	^186^
DR	Neurotrophin-4	−	Neurotrophin	The whole ER stress pathway	Protect RGCs via inhibiting ER stress	–	^241^
DR	NT4-polyamidoamine (PAMAM) electrostatic complex	−	Neurotrophin packed in nanoparticles	The whole ER stress pathway	Protect RGCs via inhibiting ER stress	–	^252^
Age-related macular degeneration (AMD)	Grape polyphenols	−	ER stress and inflammation	The whole ER stress pathway	Prevent neovascularization and protect RPE	–	^293^
AMD	Curcumin	−	ER stress	The whole ER stress pathway	Protect RPE from ER stress damage	–	^294^
AMD	ISR inhibition	−	ER stress	The whole ER stress pathway	Prevent neovascularization via inhibition of ER stress, like ATF4	–	^275^
AMD	Paeoniflorin	−	CaMKII/AMPK	The whole ER stress pathway	Protect RPE	–	^295^
AMD	Propofol	−	UPR pathway	The whole ER stress pathway	Protect RPE	–	^296^
AMD	Humanin	−	UPR pathway	The whole ER stress pathway	Protect RPE	–	^297^
AMD	Human umbilical cord mesenchymal stem cell-derived exosomes	−	miR-27b	The whole ER stress pathway	Secretion of miR-27b to inhibit fibrosis-induced ER stress	–	^299^
AMD	Human retinal progenitor cells	−	UPR pathway	The whole ER stress pathway	Supplement with neural cells to compensate ER stress-induced cell loss	–	^300^
AMD	Taurine	−	Calpain	Upregulate calpain to inhibit ER stress	Protect RPE	–	^298^
RP	4-PBA	−	UPR pathway	The whole ER stress pathway	Protect the whole retinal cell via inhibition of ER stress	Heart, lung, and kidney injury	^325,326^
RP	TUDCA	−	UPR pathway	The whole ER stress pathway	Protect the whole retinal cell via inhibition of ER stress	Studied in many neurodegenerative diseases	^325,326^
RP	Rapamycin	−	mTOR	PI3K/AKT/mTOR	Inhibit ER stress and the downstream inflammation in RPE and photoreceptors	Lifespan, cardiac disease/function, central nervous system, immune system, and cell senescence	^477^
RP	PP242	−	mTOR	PI3K/AKT/mTOR	Inhibit ER stress and the downstream inflammation in RPE and photoreceptors	In colon cancer and gastric cancer	^478,479^
RP	AICAR	−	AMPK	CAMKKβ/AMPK/mTOR	Inhibit ER stress and restore autophagy	In heart diseases	^480^
RP	Salubrinal	−	peIF2α	PERK-ATF4-CHOP	Reduce ER stress in RPE and photoreceptors	–	^308^
RP	Bilberry extract	−	UPR pathway	The whole ER stress pathway	Protect RGC loss induced by ER stress	–	^312^
RP	HSPA4L	−	PRPF31 mutant aggresomes	Protein chaperon	Transport PRPF31 mutant aggresomes to nuclear which protect RPE	Null	^481^
RP	CRIPR/Cas9	−	RHO mutation	UPR pathway	Knock out mutant RHO to reduce ER stress in photoreceptors	–	^328^
RP	siRNA	−	RHO mutation	UPR pathway	Knock out mutant RHO to reduce ER stress in photoreceptors	–	^329^
RP	Stem cell therapy	−	Stem cell differentiated into RPE cells	The whole ER stress pathway	Supplement with RPE, which prevent the retinal cell loss induced by ER stress	–	^331–333,335^
RP	Exosomes derived from mesenchymal stem cell and RPE	−	UPR pathway	The whole ER stress pathway	Prevent the whole retina from the damage of ER stress	–	^337–339^
RP	Optogenetics	−	Non-photosensitive retinal cells	The whole ER stress pathway	Converting non-photosensitive retinal cells to photoreceptors, which escape the ER stress damage resulted from RHO mutation	–	^342^
ACHM	Fenretinide	+	ATF6	ATF6 pathway	Increase the expression of ATF6 to protect cone photoreceptors	–	^353^
ACHM	AA157	+	ATF6	ATF6 pathway	Increase the expression of ATF6 to protect cone photoreceptors	–	^349^
ACHM	Rp-8-Br-cGMPS	−	cGMP	cGMP/PKG/ RyR2	Elevate of intracellular Ca^2+^ and abolish the channel upregulation in CNG channel deficiency to reduce ER stress in cone cells	In vascular and muscle field	^348^
ACHM	KT5823	−	cGMP	cGMP/PKG/ RyR2	Elevate of intracellular Ca^2+^ and abolish the channel upregulation in CNG channel deficiency to reduce ER stress in cone cells	In Marfan syndrome, thyroid cancer, and breast cancer	^348^
RP	AAV	−	CNGB3	The whole ER stress pathway	Protect cone photoreceptors	–	^356^
RP	CRISPR/Cas9	−	PDE6	The whole ER stress pathway	Protect cone photoreceptors	–	^357^
Cataracts	4-PBA	−	Protein chaperon	The whole ER stress pathway	Reduce EMT via inhibition of ER stress and reduce opacity in lens	Heart, lung, and kidney injury	^377^
Cataracts	Pentazocine	−	S1R	The whole ER stress pathway	Relieve ER stress, OS, and cell apoptosis in lens cell	–	^378^
Cataracts	Iontophoresis	−	Delivery system	The whole ER stress	Combining it with ER-targeted drugs to reduce opacity in lens	–	^379^
Uveitis	TUBCA	−	IRE1α	IRE1/NOD2 pathway and NLRP3 inflammasomes	Downregulation of ER stress via inflammation inhibition	–	^382^
Uveitis	Galectin-3	−	UPR pathway	The whole ER stress pathway	Downregulation of ER stress in BS	–	^390^
Uveitis	Mesalazine	−	UPR pathway	The whole ER stress pathway	Alleviation of inflammation via ER stress reduction in BS	–	^389^
Uveal melanoma (UM)	4-PBA	−	UPR pathway	The whole ER stress pathway	Effectively inhibit ER stress in BRAF-mutated melanoma	Heart, lung, and kidney injury	^408^
UM	Pemetrexed	+	CHOP	CHOP/ NOXA/ Mcl-1 pathway	Facilitate ER stress-induced intrinsic apoptosis	–	^410^
UM	Navitoclax	−	PERK	PERK pathway	Blocking ER stress-induced drug resistance to facilitate tumor apoptosis	–	^407^
UM	Combining targeted ER stress therapy with immunotherapy	−	UPR pathway	The whole ER stress pathway	Inhibition of ER stress, which facilitates immunotherapy effects	–	^412^
UM	Nanosystems	−	ER	The whole ER stress pathway	Directly inhibit ER stress to strengthen antitumor drugs effects	–	^412^
UM	SCD1 inhibitor	−	UPR pathway	The whole ER stress pathway andβ-catenin signaling	Production of antitumor CD8^+^ T cell by inhibiting ER stress	In human colon cancer	^412^
Corneal dystrophy	4-PBA	−	UPR pathway	The whole ER stress pathway	Reinforce ER stress and degrade TGFBIp by relieving ER stress	Heart, lung, and kidney injury	^427^
Corneal dystrophy	Melatonin	−	The UPR pathway	IRE1α/XBP1 pathway	Inhibition of ER stress to save GCD2 corneal fibroblasts	–	^431^
Corneal dystrophy	Mitochondrial ROS quencher MitoQ	−	Bip and GADD153	The whole ER stress pathway	Restore mitochondria function by inhibiting Bip and GADD153	–	^416^
Corneal dystrophy	Glafenine	−	Mutant SLC4A11	The whole ER stress pathway	Facilitate SLC4A11 folding, which inhibit ER stress-induced cell loss	–	^435^
Corneal dystrophy	Mesenchymal stem cell-derived extracellular vesicles	−	The UPR molecules	The whole ER stress pathway	Contain miRNA-targeted ER stress while facilitating cell survival in cornea	–	^436^
Myopia	Salidroside	−	HIF-1α	HIF-1α/eIF2α	Inhibition of hypoxia-induced ER stress to prevent sclera remodeling	–	^463,464^
Myopia	Formononetin	−	HIF-1α	HIF-1α/eIF2α	Inhibition of hypoxia-induced ER stress to prevent sclera remodeling	–	^463,464^
Myopia	4-PBA	−	The UPR pathway	The whole ER stress pathway	Normalize the vitreous chamber depth and retinal thickness via inhibition of ER stress	Heart, lung, and kidney injury	^459^
Myopia	TUDCA	−	The UPR pathway	The whole ER stress pathway	Normalize the vitreous chamber depth and retinal thickness via inhibition of ER stress	–	^459^
Myopia	CRIPR/Cas9	−	PERK and ATF6	PERK and ATF6 pathway	Normalize the vitreous chamber depth and retinal thickness via inhibition of ER stress	–	^458^

## Data Availability

Not applicable.
